# The Genomic Basis of Evolutionary Innovation in *Pseudomonas aeruginosa*

**DOI:** 10.1371/journal.pgen.1006005

**Published:** 2016-05-05

**Authors:** Macarena Toll-Riera, Alvaro San Millan, Andreas Wagner, R. Craig MacLean

**Affiliations:** 1 Department of Zoology, University of Oxford, Oxford, United Kingdom; 2 Institute of Evolutionary Biology and Environmental Studies, University of Zurich, Zürich, Switzerland; 3 The Swiss Institute of Bioinformatics, Lausanne, Switzerland; 4 The Santa Fe Institute, Santa Fe, New Mexico, United States of America; University of Toronto, CANADA

## Abstract

Novel traits play a key role in evolution, but their origins remain poorly understood. Here we address this problem by using experimental evolution to study bacterial innovation in real time. We allowed 380 populations of *Pseudomonas aeruginosa* to adapt to 95 different carbon sources that challenged bacteria with either evolving novel metabolic traits or optimizing existing traits. Whole genome sequencing of more than 80 clones revealed profound differences in the genetic basis of innovation and optimization. Innovation was associated with the rapid acquisition of mutations in genes involved in transcription and metabolism. Mutations in pre-existing duplicate genes in the *P*. *aeruginosa* genome were common during innovation, but not optimization. These duplicate genes may have been acquired by *P*. *aeruginosa* due to either spontaneous gene amplification or horizontal gene transfer. High throughput phenotype assays revealed that novelty was associated with increased pleiotropic costs that are likely to constrain innovation. However, mutations in duplicate genes with close homologs in the *P*. *aeruginosa* genome were associated with low pleiotropic costs compared to mutations in duplicate genes with distant homologs in the *P*. *aeruginosa* genome, suggesting that functional redundancy between duplicates facilitates innovation by buffering pleiotropic costs.

## Author Summary

Novel traits play a key role in evolution by providing organisms with access to new ecological niches. Novelty is often conspicuous at a phenotypic level, but it is difficult to determine its underlying genetic basis. To address this problem, we have studied how the bacterium *P*. *aeruginosa* evolves novel metabolic traits, such as the ability to degrade new sugars, in real-time. After 30 days of evolution we sequenced the genomes of bacteria that have evolved novel metabolic traits. We found that mutations mainly affected genes involved in transcription and metabolism. Our main finding is that novelty tends to evolve by mutations in pre-existing duplicated genes in the *P*. *aeruginosa* genome. Duplication drives novelty because genetic redundancy provided by duplication allows bacteria to evolve new metabolic functions without compromising existing functions. These findings suggest that past duplication events might be important for future innovations.

## Introduction

An evolutionary innovation is a new trait that allows organisms to exploit new ecological opportunities. Some popular examples of innovations include flight, flowers or tetrapod limbs [1,2]. Innovation has been proposed to arise through a wide variety of genetic mechanisms, including: domain shuffling [3], changes in regulation of gene expression [4], gene duplication and subsequent neofunctionalization [5,6], horizontal gene transfer [7,8] or gene fusion [9]. Although innovation is usually phenotypically conspicuous, the underlying genetic basis of innovation is often difficult to discern, because the genetic signature of evolutionary innovation erodes as populations and species diverge through time.

One way to circumvent this difficulty is to directly study the evolution of innovation in real time using microbial model systems [10,11]. The large population size and short generation time of microbes allows for rapid evolution under conditions that can be easily replicated. Samples from evolving populations can be cryogenically preserved in a non-evolving state so that evolved genotypes can be directly compared with their ancestors. Also, bacteria have compact genomes, making it possible to characterize the functional and genetic basis of adaptation [12,13]. Recent experiments using this approach have provided detailed examples of the evolution of a number of innovations [14–19], such as novel metabolic traits [15] and ecological specialization [19]. However, there is a difference between evolving a new trait (innovation) and improving an already exiting one (optimization) [17] and it remains unclear if evolutionary adaptations that require qualitatively new traits (innovations) generally have a different genetic basis than adaptations that require mere fine tuning (optimization) of an existing trait.

The objective of this study is to determine the genomic mechanisms underpinning evolutionary innovation and optimization using bacterial metabolism as a model system. To achieve this goal, we allowed populations of *P*. *aeruginosa* founded by a single clone to evolve in Biolog microtiter plates containing culture medium supplemented with 95 unique carbon sources. Crucially, the ancestral clone produces a clear bimodal pattern of growth on these carbon sources: in some of the carbon sources it grows poorly while in others it grows well. Carbon sources that support little or no growth above the carbon-free control challenge bacteria to evolve novel metabolic traits. These carbon sources can therefore be used to study evolutionary innovation. In contrast, carbon sources that allow the ancestral clone to grow to at least a moderate population density challenge bacteria to improve existing traits. These carbon sources can be used to study the genetic basis of evolutionary optimization. Following 140 generations of evolution we identified carbon sources that populations consistently adapted to. We then isolated clones from populations that evolved in these carbon sources and used whole genome sequencing of more than 80 evolved clones to determine the genetic basis of evolutionary innovation and optimization. To understand the pleiotropic consequences of innovation we used high-throughput phenotypic assays to measure the fitness of the clones evolved in a single carbon source in the 94 remaining substrates of the Biolog plate. This experimental strategy has two main benefits. First, by comparing the mutations and phenotypes observed in clones adapted through innovation and optimization it is possible to test for distinct genomic signatures associated with innovation. Second, by studying the evolution of multiple novel traits, it is possible to make general conclusions about the genetic basis of innovation.

## Results

### Defining evolutionary innovation and optimization

**Fig 1:**
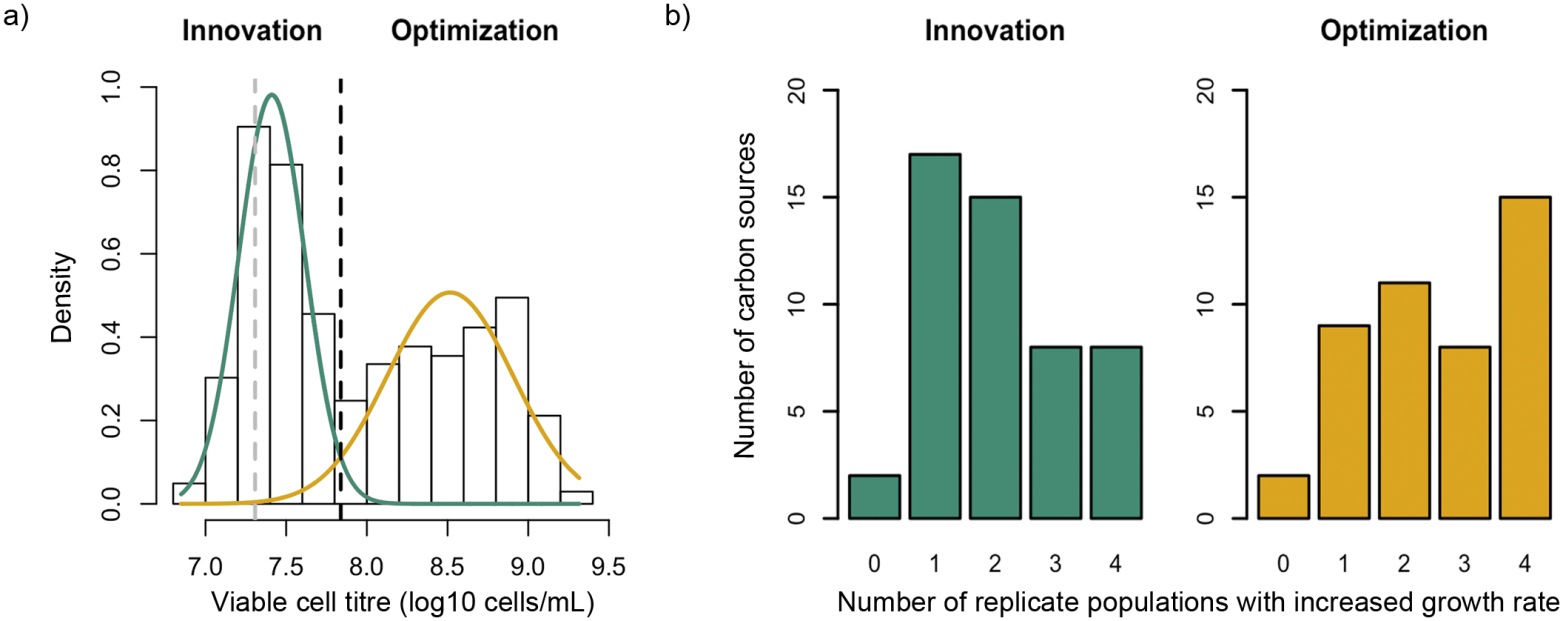
Selection for metabolic innovation and optimization. Panel **A** shows the viable cell titre of the ancestral PAO1 clone across the 95 Biolog carbon sources. The ancestral clone shows a bimodal pattern of growth. The dashed black line shows the inferred intersection between distributions of high and low growth. The dashed grey line shows growth on the negative control well that is not supplemented with any carbon sources. The solid lines show inferred distributions of viable cell titre for carbon sources that imposed selection for innovation (green) and optimization (orange). We assayed 16 replicate cultures of the ancestral strain on each substrate and the error on our estimates of viable cell density was small (average SE = 0.029 log10 cells/mL). Panel **B** shows the number of replicate populations adapted through innovation (green) and optimization (orange) with increased growth rate on each carbon source after 30 days.

We first assessed the growth of *P*. *aeruginosa* PAO1 in the 95 unique carbon sources provided by Biolog microtiter plates. Each well on a Biolog plate contains a common inorganic growth medium that is either supplemented with a unique carbon source (95 wells), or not supplemented and acts as a negative control (1 well). The parental PAO1 strain (ancestral clone hereafter) showed a clear bimodal pattern of growth in these 95 carbon sources, both in terms of viable cell titre and optical density (Fig 1A, see Materials and Methods). Some carbon sources supported very low levels of growth that were comparable to the growth observed in the negative control well; selection on these substrates challenges *P*. *aeruginosa* to evolve new metabolic traits. In contrast, other carbon sources supported good levels of growth; selection on these substrates challenges *P*. *aeruginosa* to optimize existing metabolic traits. Although this distinction is intuitive, it is necessary to formally define a threshold between innovation and optimization. To do so we fitted a mixture distribution to the viable cell titre for the 95 carbon sources. We used the point where the two distributions intersected to classify the carbon sources in two groups: innovation (carbon sources that supported poor growth, similar to the carbon-free control) and optimization (carbon sources that supported growth to high population density). This classification was also supported by optical density data (see Materials and Methods).

We evolved 4 replicate populations founded by the ancestral clone in each of the 95 carbon sources present in the Biolog microtiter plates by serially propagating cultures on 4 replicate Biolog plates for 30 daily serial transfers, which corresponds to approximately 140 generations of bacterial growth. At the end of the evolution experiment, we tested for adaptation on each of the 95 carbon sources by comparing the growth rate of the 4 replicate populations that had evolved on each carbon source to the growth rate of the ancestral clone on the same carbon source. We used growth rates to assess adaptation because they provide a higher resolution than viable cell titre, which can allow for the detection of small differences in the rate of adaptation across substrates. We note, however, that growth rate and viable cell titre measures strongly correlate (r = 0.887, P < 0.001).

Given that evolutionary innovation involves the origin of novel phenotypes, whereas optimization involves the refinement of existing phenotypes, optimization should evolve more readily than innovation. Consistent with this expectation, the proportion of populations that evolved increased growth rate was significantly lower on carbon sources that challenged bacteria to innovate as opposed to optimize existing traits (51.50% vs. 63.89%, P = 0.01, One-tailed Fisher's exact test). Moreover, the fraction of carbon sources where all 4 replicate populations evolved increased growth rate was almost 50% lower on carbon sources that challenged bacteria with evolutionary innovation as opposed to optimization (Fig 1B; P = 0.042, One-tailed Fisher's exact test).

### Identifying beneficial mutations associated with innovation and optimization

To understand the genetic basis of adaptation, we sequenced the genome of 4 independently evolved clones from carbon sources where all 4 replicate populations evolved increased growth rates. Our rationale for this sequencing strategy is as follows. Parallel increases in growth rate suggest that selection was very effective on these substrates, increasing the probability that clones from these substrates carry potential beneficial mutations. Second, by sequencing multiple clones that evolved on the same substrate it is possible to identify genes that show parallel molecular evolution. Parallelism is common in bacterial populations, and it provides a simple way to identify genes that contribute to adaptation [19–22]. Specifically, we sequenced the genomes of 84 clones from carbon sources that challenged bacteria to both innovate (8 carbon sources, 32 clones) and optimize existing traits (13 carbon source, 52 clones). The ancestral clone produces a clearly bimodal distribution of growth on these 21 carbon sources, with an approximately 10-fold difference in mean viable cell density between carbon sources where innovation as opposed to just optimization occurred (S1 Fig). We identified 143 unique mutations in the genomes of the 84 sequenced clones, amounting to a mere 1.70 mutations per clone on average (S1 Data). These were all mutations that accumulated in the course of the experiment and that were not present in the ancestral clone. Most of the mutations that we identified were SNPs (74%), but we also detected short indels (8%), large deletions (12%), and duplications (4%). The proportions of these types of mutations did not differ between clones that had adapted through innovation and optimization (S2 Fig and S1 Table; P = 0.213, Pearson’s X^2^ test). Although populations of *P*. *aeruginosa* sometimes evolve elevated mutation rates during cystic fibrosis infections [19] and during long-term selection experiments [23,24], we did not find any hypermutator strains with mutations in genes involved in DNA replication and repair, such as the methyl-directed mismatch repair pathway.

Several lines of evidence suggest that most of the SNPs that we detected were beneficial mutations. First, the vast majority (97/106) of point mutations we detected were non-synonymous (S1 Table). We only detected three synonymous mutations and two affected a gene where parallel synonymous evolution occurred, suggesting that these were beneficial synonymous mutations [25]. Thus, our estimate of the rate of substitution of non-synonymous mutations to putatively neutral synonymous mutations is 97/1. Second, the number of mutations per clone was approximately 40% higher in clones that had to adapt through innovation (2.1 mutations per clone) as opposed to clones that adapted through optimization (1.53 mutations per clone) (S3 Fig; P = 0.034, two-sample one-tailed Kolmogorov-Smirnov test). Given that the number of generations of evolution was highly similar across carbon sources (see Materials and Methods), this difference in the number of genetic changes is consistent with the idea that populations that had to adapt through innovation were exposed to stronger selection. This difference is particularly striking, given that populations that had to adapt through innovation were associated with a small population size, which should reduce the rate of fixation of beneficial mutations. Finally, parallel molecular evolution was very common: 65.73% of the mutations occurred in genes that were mutated in more than one clone (S1 Data), which is significantly greater than the amount of parallel evolution expected due to chance alone (permutation test, P < 0.001).

Gene-level parallel evolution tended to occur between replicate clones that evolved on the same carbon source, and genes that were only mutated in clones from an individual carbon source accounted for 75% of the parallelism that we observed. Interestingly we found parallel evolution in all 4 replicate clones that evolved on 5 carbon sources (L-alanyl-glycine, glycyl-l-glutamic acid, L-serine, D,L- α glycerol phosphate, and glycerol), involving 24 mutations. In every case, parallel evolution on these substrates involved transcriptional regulators. Recent work in the closely related bacterium *P*. *fluorescens* suggests that parallel evolution by mutations in transcriptional regulators is common because it provides an efficient mechanism to translate genetic variation into phenotypic variation [26]. This may explain why parallel regulatory evolution was very common on some substrates. Rigorous test of this idea is outside the scope of this paper and it would require further experimental work, as in [26]. We also observed higher-order parallel evolution involving different genes that act in the same operon. Parallelism by definition becomes more common as the scale at which it is measured increases; for example, parallelism is necessarily more common when it is measured at the level of genes than at the level of nucleotides. However, it is difficult to objectively measure parallelism above the level of the gene at a genome-wide scale, especially given the large number of genes of unknown function in the *P*. *aeruginosa* genome, and we therefore, focused our analysis of parallelism at the level of genes.

### Functional changes associated with innovation and optimization

Like many free-living bacteria, the genome of *P*. *aeruginosa* is made up almost entirely of protein coding sequences (89.4% coding DNA). Because innovation involves the origin of novel phenotypes, it is reasonable to expect that innovation should be associated with more radical changes to proteins. The vast majority of mutations that we observed were non-synonymous substitutions in protein coding regions (S1 Table), but the relative frequency of radical amino acid substitutions did not differ significantly (Z-test; P = 0.84) between evolutionary innovation (n = 21; 53.8%) and optimization (n = 28; 56.8%). Short insertions and deletions (indels) that introduce frameshifts can also produce radical changes to proteins. However, we only found 6 indels that introduced frameshifts, making it impossible to test for a difference in the frequency of indels observed under innovation (n = 4) and optimization (n = 2). In summary, innovation and optimization did not leave distinct signatures on the structure of proteins in our experiments.

**Fig 2:**
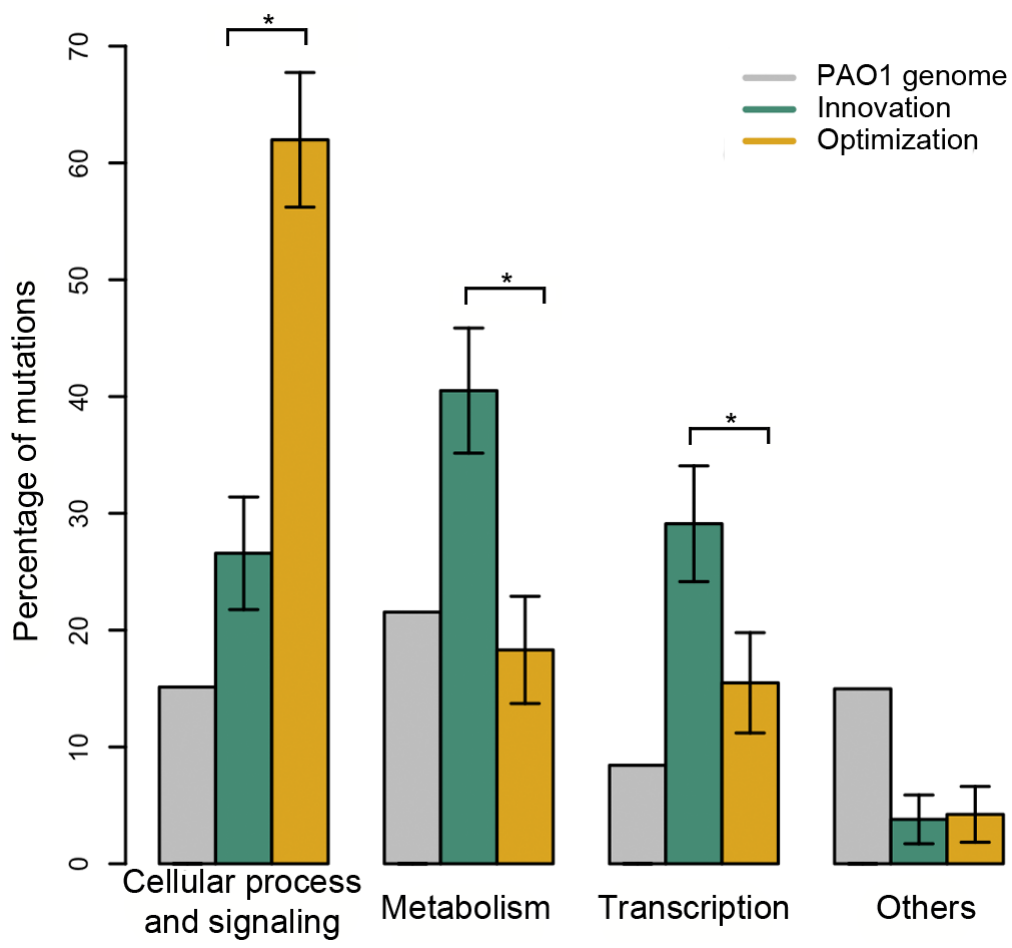
Functional basis of innovation and optimization. This figure shows the mean percentage of mutations (+/- s.d) found in different COG categories in clones that had to adapt through innovation (green) and optimization (orange). The percentage of genes in each COG category in the *P*. *aeruginosa* PAO1 genome is also shown as a reference (light grey). * indicates significant differences

To gain further insights into the mechanistic basis of adaptation, we compared the functional roles of genes carrying mutations in clones evolved through innovation and optimization. Changes in the regulation of gene expression have been proposed to play an important role in evolutionary innovation [16,27,28]. Mutations in regulatory genes were common, and in many cases these mutations could be clearly linked to metabolic traits that were under selection (S2 Table). For example, adaptation to L-serine repeatedly evolved by non-synonymous mutations in a transcription factor (PA2449) that regulates the expression of genes involved in serine metabolism [29]; similarly, acquiring the ability to metabolize L-Alanyl-Glycine repeatedly evolved by mutations in *pdsR*, a repressor of a di-peptide and amino acid transport operon. We found that the proportion of mutations in genes involved in transcription was greater in clones from populations that had to adapt through innovation as opposed to optimization, supporting the idea that altered gene expression is an important feature of innovation (Fig 2, S3 Table; P = 0.036, One-tailed Fisher’s Exact Test).

Intergenic mutations also have the potential to change gene expression, for example by altering transcription factor binding sites [16]. For example, evolution in both L-aspartic acid and L-glutamic acid resulted in parallel substitutions in the promoter region of a *P*. *aeruginosa* homolog (PA5479) of a *Bacillus subtilis* L-aspartate and L-glutamate transporter [30]. Similarly, one clone evolved in D-Serine and one clone evolved in Glycerol have, respectively, a SNP upstream D-Amino acid dehydrogenase (PA5304) and a SNP upstream a glyceraldehyde-3-phosphate dehydrogenase (PA2323). However, intergenic sequences make up only 10.6% of the *P*. *aeruginosa* genome, suggesting limited potential for adaptation by regulatory mutations in non-coding sequences. Consistent with this idea, we detected only a very small number of intergenic mutations in clones evolved through innovation (n = 4) and optimization (n = 6), making it impossible to rigorously test the role of intergenic mutations in innovation.

It is difficult to make a priori predictions regarding associations between other functional categories of genes and innovation, but we found that innovation was also preferentially associated with mutations in metabolic genes (Fig 2, S3 Table; P<0.01, One-tailed Fisher’s exact test), whereas optimization was associated with mutations in genes involved in cell processes and signalling (Fig 2; P<0.01, One-tailed Fisher’s exact test).

Recent work in experimental evolution has focused on understanding the detailed molecular mechanisms by which individual beneficial mutations increase fitness (e.g.:[15,18,25,31,32]), and this work has made an important contribution to a broader functional synthesis in evolutionary biology [33]. We found that 46% of all mutations occurred in genes that were only mutated on a single carbon source and 83.6% of the mutated genes were only mutated in one carbon source, suggesting that substrate-specific adaptation was a key driver of evolution in this experiment. In many cases, mutations in these genes can be putatively linked to the metabolism of the carbon source that populations evolved on, and this was particularly the case for genes involved in transcription and metabolism and among clones that had to adapt through innovation (S2 Table). At the same time, we also found mutations in a small fraction of genes (16.4%) across multiple carbon sources. As an extreme example we found a gene (PA1561) involved in aerotaxis, mutated 16 times across 10 substrates, suggesting that mutations in this gene represent a general adaptation to Biolog plates. Unfortunately, it is impossible to precisely measure the substrate specificity of the mutations that we detected without carrying allelic replacement experiments to generate strains carrying single mutations. In S2 Table we provide a list of the mutations that occurred on each carbon source and their putative role. However, rigorously determining the biochemical basis of the fitness advantages conferred by individual mutations is outside the scope of this article, as our goal is to understand the genetic mechanisms of evolutionary innovation, and not the biochemical basis of novel metabolic pathways. Moreover, achieving a detailed functional understanding of adaptation in this system would be incredibly challenging given the diversity of selective pressures that we imposed and the diversity of mutations that we observed.

### Gene duplication and innovation

Gene duplication is a major source of evolutionary innovation [6,34], and some elegant studies show that it can facilitate adaptation in bacterial populations [35,36]. We detected six cases of de novo gene duplication. Every case involved parallel duplications, suggesting that duplication was adaptive. Strikingly, all four clones that adapted through innovation on glycyl-L-glutamic acid evolved independent duplications of a 5.6 Kb region that contains an operon (PA4496-PA4500) involved in di-peptide and amino acid transport [37] (S4A Fig). Using information on the frequency of SNPs in the sequenced clones, we were able to re-construct the evolutionary history of these duplications. Adaptation to glycyl-L-glutamic acid evolved via a repeatable two-step process. The first is a missense or nonsense mutation in the repressor of the operon, *psdR* (PA4499). The second is a tandem duplication of the operon, most likely as a result of homologous recombination between the flanking sequences of the operon (S4B Fig). The inactivation of the repressor plus the duplication of the operon probably results in increased expression of this operon. We were able to infer the chronology of this adaptation because all reads supported the novel mutations in the *pdsR* gene, as we would expect if duplication followed mutation. This multi-step process of potentiating mutations that alter the regulation of an operon, followed by adaptive gene amplification, is very similar to a previously described mechanism for the evolution of citrate utilization in *Escherichia coli* [15]. We also found large (> 300 genes, >5% of genome) duplications in two of the four clones that adapted through optimization on hydroxyl-L-proline. These duplications overlapped in a large region comprising most of their genes (262 genes, ≈288 Kb). This overlap suggests that the duplications were adaptive, but their large scale makes it difficult to infer exactly why. Overall, the limited incidence of duplication in clones that adapted through either innovation (n = 4) or optimization (n = 2) suggests that de novo duplication is not frequently involved in metabolic innovation. This result is consistent with recent work showing that de novo duplication makes only a minor contribution to adaptation to gene loss in *E*. *coli* [16] and yeast [38].

In addition to the origin of novel duplicate genes, the divergence of already existing duplicate genes in the *P*. *aeruginosa* genome can also play a key role in evolutionary innovation [39]. To test its importance in our experiment, we classified *P*. *aeruginosa* genes into duplicates and singletons using a clustering method based on Blast similarity searches. Sequence similarity in bacterial genomes can arise as a consequence of gene duplication of existing genes in the genome, which produces paralogs that are similar as a result of shared ancestry. Alternatively, bacteria can acquire new genes that are similar to existing genes in the genome by horizontal gene transfer. In practice it is very difficult to distinguish between these two mechanisms for the origin of novel genes, and Lerat and colleagues have proposed the term synologs to describe homologous genes in bacterial genomes [40].

**Fig 3:**
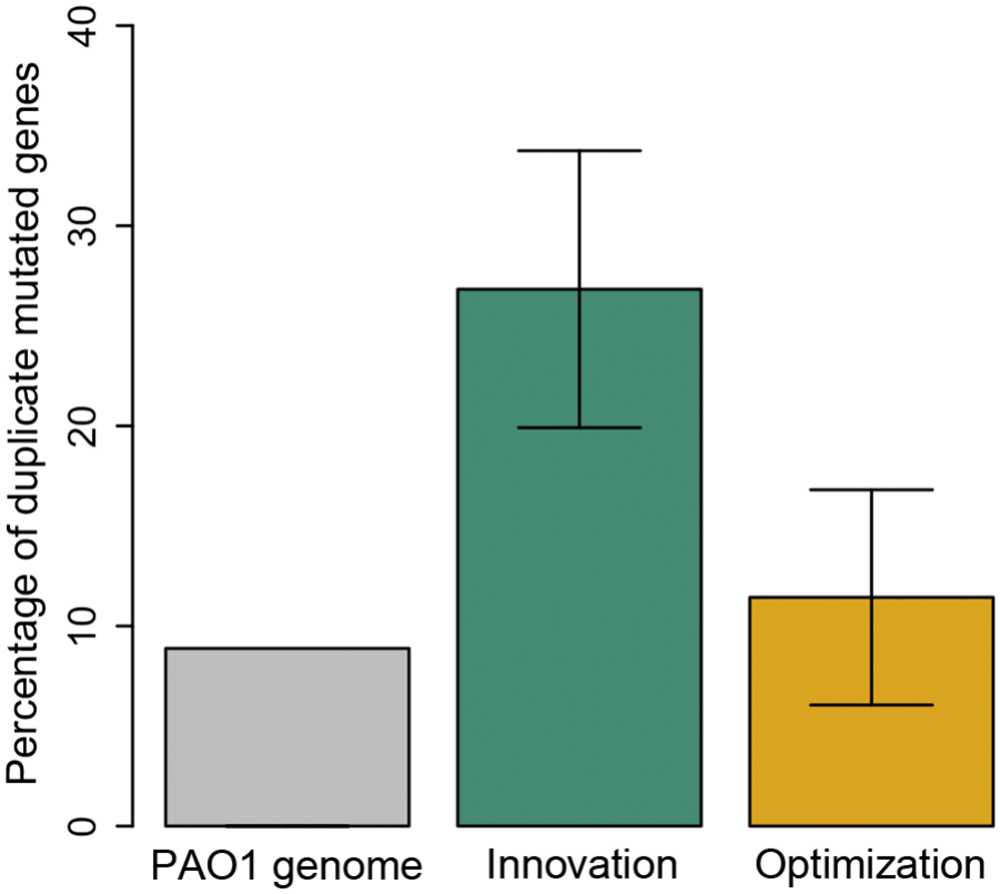
Duplication is associated to evolutionary innovation. This figure shows the percentage (+/- s.d) of mutated genes in clones that had evolved through innovation (green) and optimization (orange) that are duplicates in the *P*. *aeruginosa* genome. The percentage of duplicate genes in the *P*. *aeruginosa* genome is shown as a reference (light grey).

We found that clones that adapted through innovation acquired more mutations in existing duplicate genes than expected due to chance alone based on the frequency of duplicate genes in the *P*. *aeruginosa* genome (Fig 3, P<0.01, Pearson’s X^2^ test). In contrast, the frequency of mutations in duplicate genes in clones that adapted through optimization was indistinguishable from the frequency of duplicates in the *P*. *aeruginosa* genome (S4 Table; P>0.05, Pearson’s X^2^ test). We repeated this analysis using a broad range of similarity cut-offs to identify duplicate genes (see Materials and Methods). Our results remained robust, we consistently detected an enrichment of mutations in duplicate genes in clones that adapted through innovation, irrespective of the cut-offs used to identify duplicates (S5 Fig and S4 Table). This result suggests that the divergence of existing duplicates plays an important role in the ability to evolve novel metabolic phenotypes. We re-emphasize, however, that this analysis does not distinguish between duplicate genes that arose due to horizontal gene transfer and spontaneous duplication.

### Pleiotropic costs of innovation and optimization

What constrains the evolution of metabolic innovations that could allow *P*. *aeruginosa* to expand its ecological niche? One possible answer is that fitness costs associated with novel metabolic traits may impose a trade-off that limits metabolic innovation [41–43]. To test this hypothesis, we measured the growth of 2 of the sequenced clones from each carbon source across the 94 alternative carbon sources present on a Biolog plate, and we compared it to the growth of the ancestral clone on each carbon source. We did a total of 4750 growth assays (S6 Fig) and we established conservative criteria to infer positive or negative pleiotropy. Because our evolved clones carried only a small number of beneficial mutations (1.70 mutations per clone on average), we can be confident that altered growth on alternative carbon sources reflects the pleiotropic side-effects of beneficial mutations. However, we cannot entirely rule out the possibility that some clones carried conditionally neutral mutations that spread by hitch-hiking with beneficial mutations, but the scarce number of synonymous mutations suggests that conditionally neutral mutations are infrequent.

**Fig 4:**
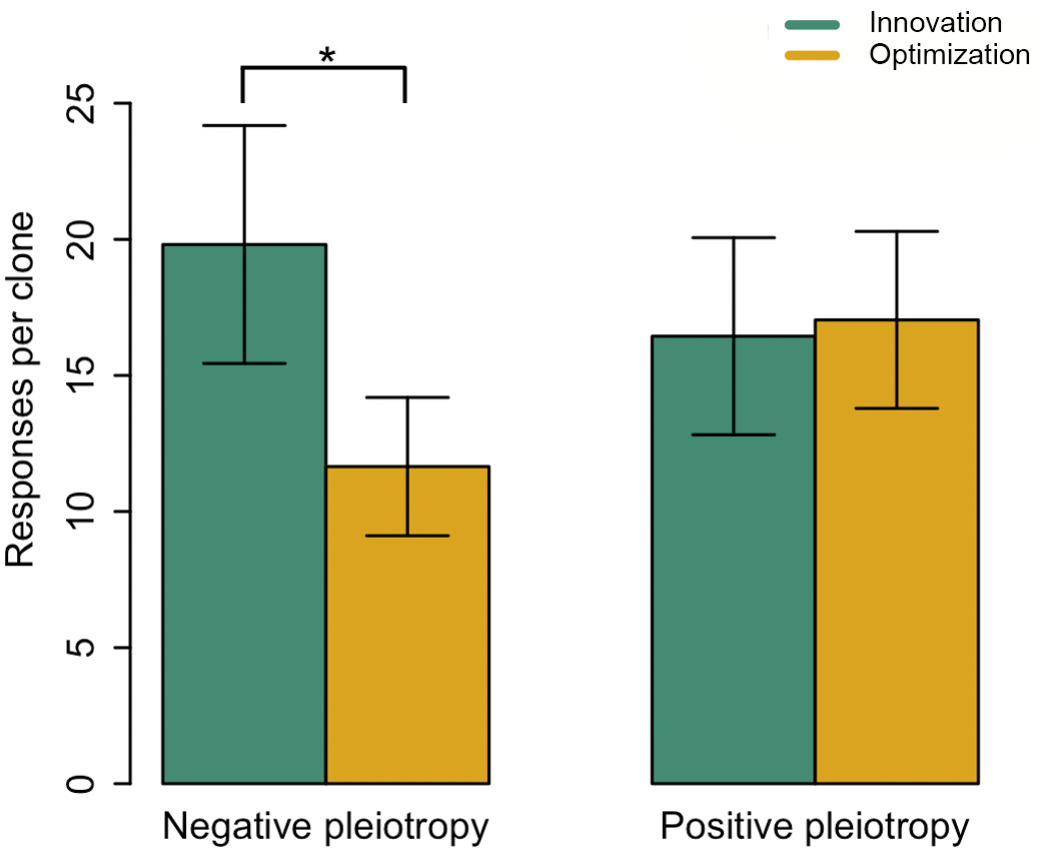
The cost of innovation. This figure shows the mean (+/- s.d) number of positive (increased growth) and negative (decreased growth) pleiotropic effects associated with evolutionary innovation (green) and optimization (orange). * indicates significant differences

Adaptation was associated with pleiotropic costs, because evolved clones showed reduced growth on an average of 14.76 carbon sources that could be used by the ancestral clone. However, the pleiotropic cost of innovation was 70% greater than the pleiotropic cost of optimization, which is consistent with the idea that pleiotropy constrains innovation (Fig 4, S5 Table; P<0.01, Pearson’s X2 test). The precise mechanistic causes of negative pleiotropy are difficult to determine [44] without measuring the effects of the individual mutations that contributed to adaptation in our system. However, the association between evolutionary innovation and mutations in regulatory and metabolic genes suggests that mutations in both of these categories of genes are likely candidates to explain negative pleiotropy. While these observations show that both innovation and optimization have costs, not all pleiotropy may be negative. Surprisingly, we found that positive pleiotropy—where an evolved population shows increased growth on one or more alternative carbon sources—was just as common as negative pleiotropy. The frequency of positive pleiotropy did not differ between clones that adapted through innovation and optimization (Fig 4, S5 Table; P = 0.61, Pearson's X2 test).

### Genetic redundancy minimizes costs of evolutionary innovation

Clones that adapted through innovation were enriched in mutations in duplicated genes and paid higher pleiotropic effects than clones that adapted through optimization. This observation is counter-intuitive, because we would expect that mutations in existing gene duplicates should be associated with low pleiotropic costs, given that the other copy of the duplicate may provide functional backup for the mutated copy. To explore this counter-intuitive observation further, we compared the pleiotropic costs expressed by clones carrying mutations in duplicates genes that have close or distant homologs in the *P*. *aeruginosa* genome. This analysis is motivated by the assumption that functional redundancy between duplicate genes decays as they diverge from each other. Interestingly, we found that clones carrying mutations in genes that have close homologs have a lower pleiotropic cost than clones without mutations in genes with close homologs (Table 1, S6 Table; P<0.01, Pearson's X^2^ test). In contrast, we see the opposite pattern in distant homologs: The pleiotropic cost of clones with mutations in genes with distant homologs is higher than that of clones without mutations in distant homologs (Table 1, S6 Table; P<0.01, Pearson's X^2^ test). Collectively, these results support the idea that redundancy between duplicates minimizes the cost of innovation.

**Table 1:** Gene duplication provides functional backup and reduces the cost of innovation.

	N clones	Mean cases of + pleiotropy	Mean cases of—pleiotropy
**Duplicates with close homologs**			
Clones carrying mutations in genes with close homologs	9	22.222 (SE = 4.564)	9.777 (SE = 3.643)
Clones not carrying mutations in genes with close homologs	33	15.333 (SE = 3.897)	16.121 (SE = 2.787)
**Duplicates with distant homologs**			
Clones carrying mutations in genes with distant homologs	13	10.615 (SE = 2.784)	23.154 (SE = 4.348)
Clones not carrying mutations in genes with distant homologs	25	17.64 (SE = 3.166)	12.400 (SE = 2.830)
**All duplicates**			
Clones carrying mutations in duplicates	22	15.364 (SE = 2.999)	17.682 (SE = 3.242)
Clones not carrying mutations in duplicates	20	18.400 (SE = 3.897)	11.550 (SE = 3.322)

Average number of positive and negative pleiotropic effects for clones carrying mutations in genes with close and distant homologs compared to clones carrying mutations in non-duplicated genes. Standard error of the mean (SE) is indicated in parenthesis.

## Discussion

Microbiologists have known for a long time that bacteria can evolve novel metabolic traits in the laboratory [45], and we have taken advantage of the experimental tractability of microbial metabolism to study evolutionary innovation at a broad scale using high-throughput experimental methods coupled to whole genome re-sequencing. This approach provides the opportunity to study the generality of evolutionary outcomes under a range of selective conditions [16,38]. Using this approach, we have shown that there are significant differences in the genomic basis of metabolic innovation and optimization in *P*. *aeruginosa*. Opportunistic pathogens, such as *P*. *aeruginosa*, encounter a novel niche when they establish long-term infections in human hosts, and altered metabolism plays a role in evolutionary transition to specialization on a pathogenic lifestyle [46]. Understanding the causes of evolutionary innovation may, therefore, contribute to our ability to predict the evolution of host-specialization in pathogenic bacteria.

At a functional level, we found that both innovation and optimization are predominantly driven by substitutions in proteins, which is hardly surprising given that the genome of *P*. *aeruginosa* is made up of 90% coding DNA. Interestingly, innovation and optimization leave similar signatures in proteins, and we did not find any evidence of an excess of radical substitutions associated with innovation. In contrast, we found profound changes in the functional roles of genes that contributed to innovation and optimization. Specifically, we found that innovation is associated with mutations in transcription regulators and metabolic genes. Changes in the expression of existing metabolic pathways that have a basal or underground ability to metabolize novel compounds and changes in the structure of metabolic enzymes that increase their activity towards novel substrates could be involved in the origin of innovations. Importantly, previous studies have provided detailed examples of how both of these mechanisms can lead to evolutionary innovation in bacteria [15,45,47–49].

One of the main results of our study is that mutations in pre-existing duplicate genes in the *P*. *aeruginosa* genome play an important role in metabolic innovation, but not optimization. It is important to recall that we identified duplicate genes based on sequence similarity, and not necessarily common ancestry. Importantly, this method does not distinguish between duplicates that arise via spontaneous duplication (paralogs) and horizontal gene transfer, but irrespective of the origins of the duplicates, duplication is expected to result in genetic and functional redundancy [50]. Why are duplicates so important for innovation? Our results show that evolving new metabolic traits is associated with pleiotropic costs. This is not surprising given that innovation is associated with mutations in genes involved in transcription and metabolism. Trade-offs between evolving novel metabolic pathways and maintain existing ones may therefore constrain innovation. How can this obstacle be overcome? Carrying duplicate genes produces redundancy, and this redundancy can potentiate innovation through neo-functionalization [4–6,51]. The presence of an extra gene copy with functional overlap increases mutational robustness and this increase facilitates the exploration of novel gene functions while the other copy maintains its ancestral function [52–54]. The importance of gene duplication for mutational robustness is still debated [54–60]. Our results support its importance. We find that mutations in genes that have a close homolog in the genome tend to be associated with lower costs than mutations in duplicate genes that have distant homologs. Therefore, our experiments provide evidences of a link between duplication, robustness, and evolvability in *P*. *aeruginosa*.

In contrast to eukaryotes, most new genes in **γ**-proteobacteria, including *P*. *aeruginosa*, are acquired by horizontal gene transfer, and not by gene duplication [40]. For example, we identified approximately 10% of genes in the *P*. *aeruginosa* genome as being pre-existing duplicates, whereas Lerat and colleagues [40] estimated that only about 1% of genes in **γ**-proteobacteria genomes arise by duplication. This discrepancy suggests that pre-existing horizontally acquired genes are likely to have played a key role in evolutionary innovation in our experiment. Horizontal gene transfer has mainly been viewed as an important source of evolutionary innovation by providing bacteria with access to a very wide pool of genes that confer novel and important phenotypes, such as antibiotic resistance in pathogenic bacteria [8,61,62]. Our results suggest that the horizontal acquisition of functionally redundant genes may also play a key role in evolutionary innovation by providing bacteria with increased genetic robustness to mutations that generate novel phenotypes.

Although redundant duplicate genes provide a genetic substrate for innovation, it is well established that acquiring new genes as a result of horizontal gene transfer or gene duplication, carries a fitness cost in bacteria [31,36,63–66]. Owing to this cost, newly acquired genes tend to be lost from bacterial populations unless gene acquisition, per se, is beneficial [6,36] or because addiction genes, such as toxin-antitoxin systems, select against the loss of acquired genes [67]. Indeed, fitness costs may explain why we observed so few instances of *de novo* duplication. Selection against newly acquired redundant genes in the short term may therefore limit the long-term ability of bacteria to evolve novel phenotypes. A key goal for future work will be to understand how this tension between fitness and evolvability arising from gene acquisition is resolved.

## Materials and Methods

### Bacterial strains and growth conditions

In this study we used two bacterial clones, *P*. *aeruginosa* PAO1 (PAO1-wt) and a *P*. *aeruginosa* PAO1 containing the *luxCDABE* operon (PAO1-lux) for luminescence production inserted in a neutral site in the bacterial chromosome (PAO1::mini-Tn7-p*LAC*-*lux*) [68]. The two clones are genetically identical except for the lux operon. We cultured the strains in Biolog GN2 96-well plates (Biolog, USA), in a final volume of 125 μl of M9 broth (Fischer Scientific, USA) per well, at 37°C and without shaking. Biolog GN2 plates contain 95 different carbon sources plus a negative control well.

### Experimental evolution

We first cultured bacteria on LB agar plates (Fischer Scientific, USA) at 37°C to obtain isolated colonies. We initiated the evolution experiment by inoculating a single colony in each well of a 96 well-plate containing LB broth. Alternating wells contained PAO1-wt and PAO1-lux clones to control for possible cross-contamination occurring during the experiment. We build two plates with two alternative patterns: well A1 containing PAO1-wt (plate A) and well A1 containing PAO1-lux (plate B). We incubated the 96 well-plate at 37°C and 225 rpm overnight and we started the experimental evolution with two replicates from each plate (plate A1, plate A2, plate B1, plate B2).

We propagated these four plates independently in Biolog GN2 plates for 30 transfers. Specifically, we transferred 5μl of bacterial culture every day into a fresh Biolog plate with 120 μl M9 broth in each well, allowing approximately 4.6 generations per day (log_2_ of the dilution factor) and a total of 140 generations during the experiment. Note that this calculation estimates equal bacterial density after incubation in a given well (carbon source) over the entire experiment. An increase in the maximum cell density in a well over the 30 days due to adaptation to the carbon source will slightly increase the total number of generations (in our experiment, the most extreme example went up to 143 generations). These differences are not big enough to significantly affect the mutation supply in the different environments over the experiment. We assessed OD and luminescence every day using a BioTek Synergy H4 plate reader (Biotek Instruments, UK). We incubated the plates at 37°C for 24 hours with no shaking.

### Ancestor's initial adaptation to Biolog carbon sources

To assess how well adapted the ancestral strain was to each of the carbon sources in a Biolog GN2 plate, we estimated the number of viable cells in each well after a 16 hour incubation of the parental PAO1 strain. To this end, we first streaked out freezer stocks of wild type ancestors to isolate single colonies, and cultured a single colony in 3 ml LB tubes overnight at 37°C with continuous shaking (225 rpm). On the next day we diluted 5 μl of the overnight culture into 20 ml of M9 broth, and inoculated a Biolog GN2 plate with 125 μl of the dilute cell suspension per well. We incubated the culture for 16 hours at 37°C without shaking, diluted it ten-fold, and measured cell viability using the BacTiter-Glo Microbial Cell Viability Assay (Promega, USA). We assessed luminescence produced in this assay using a BioTek Synergy H4 plate reader (BioTek Instruments, UK) for a total of 16 technical replicates per carbon source. We calculated the final bacterial density in each well with an in-house R script. The numbers of viable cells in the different wells followed a clear bimodal distribution (Fig 1A). We fitted a mixture distribution to the data using the mixtool package in R [69]. We used the point at which the two distributions intersected (107.84 bacteria/mL) to classify Biolog carbon sources into two groups, one in which the ancestor grew very poorly (and required innovation to adapt during the evolution experiment), and another where the ancestor grew well (and required only optimization) (Fig 1A, S1 Fig). To strengthen our classification we also used OD data obtained before performing the BacTiter-Glo Microbial Cell Viability Assay. We performed a hierarchical clustering analysis using viable cells and OD data (S7 Table) using the hclust package in R [70]. Three clear groups clustered together (S7 Fig): carbon sources classified as innovation using the intersection of the two distributions, carbon sources classified as optimization using the distribution intersection and a last group containing three carbon sources that according to the distribution intersection were classified as optimization (N-acetyl-D Glucosamine, Sebacic Acid and Hydroxy-L-Proline). These three carbon sources were clustered together because they have lower OD values than the other carbon sources classified as optimization. We decided to maintain our classification and categorize them as optimization because the BacTiter-Glo Microbial Cell Viability Assay has a better resolution than OD. However, the main results of the paper did not change if we classify these three carbon sources as innovation.

### Assessing adaptation using growth rates

To identify which populations had adapted after 30 days of evolution we measured growth curves both for ancestor and final populations at the population level. We used growth rates to determine differences in the fitness of these populations because the growth rate provides a higher level of resolution than final cell density in the population. This higher resolution is needed because we are now comparing the changes in fitness in a given carbon source over the 30 days of experiment, which are subtle (compared to the differences observed for the parental strain over the 95 different carbon sources). To this end, we cultured freezer stocks of both populations in LB broth (200 μl/well, 96-well plates) overnight at 37°C, 225 rpm. The next day we diluted the bacteria 1000-fold in M9 broth, and cultured them overnight in Biolog GN2 plates at 37°C (125 μl/well). The following day we performed another passage, diluted the culture 1:1000 in fresh Biolog G2 plates using fresh M9 broth (final volume, 125 μl/well), and measured growth rate for 16 hours using a BioTek Synergy H4 plate reader (BioTek Instruments, UK) at 37°C with no shaking. We computed the maximum growth rate with the software Gen5 2.00 (BioTek Instruments, UK). Then, from the populations in which we observed an increase in growth rate, we isolated a single clone from the freezer stock using LB agar plates (Fischer Scientific, USA) and repeated the same protocol to ensure that we found the same behaviour at clone level. We stored frozen stocks of these clones. Subsequently, we sequenced the genomes of selected clones, i.e., clones evolved in carbon sources that fulfil the following condition: The maximum growth rate for the final population (and clone) was higher than that of the ancestral population for all four replicates of populations evolved in that carbon sources.

### Whole genome sequencing analysis

We performed DNA extractions from clones cultured in 3 ml LB broth (Fischer Scientific, USA) that had been incubated at 37°C with 225 rpm shaking overnight, using the Qiagen Dneasy Blood and Tissue Kit (Qiagen, Inc., Chatworth, California, USA) and the Promega Wizard Genomic 4 DNA Purification Kit (Promega, UK). We quantified DNA using the QuantiFluor dsDNA system (Promega, Madison, WI, USA) following manufacturers' instructions.

We conducted library preparation and sequencing (using HiSeq2000 and 100-bp-paired end reads) at the Wellcome Trust Centre for Human Genetics, University of Oxford. We sequenced 88 genomes, i.e., 2 PAO1-lux ancestral strains, 2 PAO1-wt ancestral strains, and 84 evolved clones. We analyzed sequencing data using a pipeline developed in-house, as previously described in San Millan *et al*. 2014 [71], and mapped filtered reads to our reference genome, which is P. aeruginosa PAO1 (NC_002516.2) with the insertion of the phage RGP42 (GQ141978.1). We analyzed only those mutations that had accumulated during the experiment and that were not present in our ancestral strains at the start of the experiment. Note that each evolved clone was compared to the specific ancestral clone from which it was derived (i.e. either PAO1-wt or PAO1-lux).

The reads generated in this work have been deposited in the European Nucleotide Archive database under the accession code PRJEB12874.

### Experimental evaluation of pleiotropy

To elucidate how frequently adaptation to a specific carbon source affects growth in other carbon sources, we performed an experiment at the level of individual clones, using two evolved clones per carbon source and four wt clones as controls. Specifically, we tested those clones whose growth rate had increased during the experiments (the same clones that we used for the whole genome sequencing). We inoculated each clone that had adapted to a particular carbon source in the 95 carbon sources of a Biolog plate. To this end, we cultured each frozen clone and four wt clones in 3 ml LB tubes overnight at 37°C with continuous shaking (225 rpm), diluted 5 μl of the overnight culture on the next day into 20 ml of M9 broth, and inoculated a Biolog GN2 plate with 125 μl of the dilute cell suspension per well. We incubated the plate for 16 hours at 37°C without shaking. Subsequently, we diluted each culture 10-fold, and measured cell viability in 384-well black plates, using the BacTiter-Glo Microbial Cell Viability Assay (Promega, UK), following manufacturer's instructions. We assessed luminescence produced in the BacTiter-Glo assay using a FLUOstar OPTIMA plate reader (BMG Labtech, UK).

We developed an R script to calculate the number of doublings bacteria experience in each well of the Biolog plate correcting for the number of doublings in the negative control well (number of effective doublings). We used the values obtained from 4 wt replicate controls to calculate the 95% confidence interval of number of effective doublings for each well. Then, to assess the pleiotropic effects that the adaptation to a particular carbon source had in the 94 remaining carbon sources, we checked if the number of doublings in each carbon source for that particular clone fell outside the 95% confidence interval calculated using the wt measurements. If it fell outside the 95% confidence interval we counted it as a positive pleiotropic effect in that specific carbon source if the number of doublings were higher than for the wt or as negative pleiotropic effect if the number of doublings were lower than for the wt.

### Type of amino acid replacement

We classified those mutations that involve amino acid replacements as radical if they were associated with a change of polarity group (polar: C, N, Q, S, T and Y; nonpolar: A, F, G, I, L, M, P, V and M; positively charged: H, K, and R; and negatively charged: D and E) and as nonradical when the replacement did not imply a change of polarity group.

### Gene duplication analysis

To classify *P*. *aeruginosa* PAO1 genes into duplicates and singletons, we used BLASTclust (ftp://ftp.ncbi.nih.gov/blast/documents/blastclust.html). BLASTClust is a stand-alone program used to cluster proteins based on pairwise matches using the BLAST algorithm. We considered as singletons all proteins that formed a cluster whose only member was the protein itself, and as duplicates when the cluster contained more than one protein [72,73]. Note that this method does not distinguish between duplicates originated by gene duplication of existing genes in the genome and horizontal gene transfer. To ensure the robustness of the classification we used 10 different cut-offs of minimum length coverage and percentage of identical residues. The results remained robust to the different cut-offs used (S4 Table). Higher values of length coverage and residue identity are more likely to indicate close homology [74]. Moreover, it has been previously suggested that values of 53% coverage and 31% identity are enough to detect homology in duplicates [73,75]. For these reasons and also to ensure having enough number of genes classified in each category, we used the cut-off 70 coverage and 50 identity as an indicator of close homology and the cut-off 50 coverage and 40 identity as an indicator of distant homology.

We experimentally validated observed duplications after 30 days of evolution in glycyl-L-glutamic acid and hydroxyl-L-proline by PCR-amplifying the edges of the duplication and Sanger sequencing the products to confirm the results (S8 Table and S4A Fig).

### Parallel evolution and permutation test

We assessed statistically if the number of parallel mutations observed in our dataset is higher than the expected by chance. We randomly selected a total of 143 positions in the coding region of the *P*. *aeruginosa* PAO1 genome, corresponding to the total number of identified mutations. We then recorded the proportion of loci located in genes with more than one randomly selected position and repeated the procedure 1,000 times. As a result, we generated the expected distribution by chance (mean = 0.04, sd = 0.02). We obtained an empirical estimation of the P-value as the proportion of permutations yielding a value more extreme than the observed in our dataset.

### Statistical analyses

We performed all statistical analyses and produced all graphics using R [61].

## Supporting Information

S1 Data List of mutations identified in the 84 sequenced clones. (XLS)

S1 Fig Population density of the ancestral *P*. *aeruginosa* PAO1 strain in the carbon sources where the experimental populations increased growth over time. Panel **a** shows the substrates where the ancestral strain grew poorly (innovation) and panel **b** shows the substrates where the parental PAO1 was able to grow efficiently (optimization). Growth was calculated as viable cell titre (log10 cells/mL), 16 replicates per carbon source.
(TIF)

S2 Fig Types of mutations identified in clones that had to adapt through innovation compared to clones that had to adapt trough optimization. Large deletions > 30 bp.
(TIF)

S3 Fig Average number of mutations per carbon source in clones that had to adapt through innovation (panel a) compared to clones that had to adapt through optimization (panel b). (TIF)

S4 Fig Genetic bases of the adaptation to glycyl-L-glutamic acid in *P*. *aeruginosa* PAO1. Panel **a**, The schematic diagram shows the genetic environment of the operon (red arrows) mutated in all the four clones that adapted to glycyl-L-glutamic acid. This operon is involved in di-peptide and amino acids transport. The reading frames for genes are shown as arrows, with the direction of transcription indicated by the arrowhead. PsdR is a transcriptional regulator that represses the expression of mdpA (metallopeptidase) and ddpA3 (dipeptide binding protein involved in the internalization of glycyl-L-glutamic acid), as indicated by the red minus symbols in the figure. Yellow ellipses indicate the positions of the mutations in psdR and the changes in the predicted protein sequence are described below them (“del 76 nt” indicates a deletion of 76 nucleotides). Dashed lines indicate the duplicated region between ddpA1 and ddpA3. The numbers in the left and right above the sequence indicate the genomic location in the *P*. *aeruginosa* PAO1 genome (NCBI taxonomy ID: 208964). We used Primer 1 (5’->3’,CGCGAGGCCGGGAAGGACCTT) and Primer 2 (5’->3’, TTGCCATGGCCCATAAGGCC) to confirm the duplications. The picture below the sequence shows the result of the agarose gel electrophoresis of the PCR products using primers 1 and 2. The first lane contains the molecular weight marker; the next two lanes are two negative controls using water and the DNA from the parental PAO1 strain as template for the PCR reaction (note that both reactions are negative). The remaining lanes show the result of the PCR using DNA samples from the four PAO1 clones tested in this experiment, which had evolved in the presence of glycyl-L-glutamic acid as the sole carbon source for 30 days (F12 A1, F12 A2, F12 B1, F12 B2). Note that the PCR reaction for all the clones is positive. We sequenced the PCR products confirming the results. Panel **b**, Tandem duplication as a result of homologous recombination. Nucleotide alignment between the 5' region of the genes dppA1(PA4496), dppA3 (PA4500) and an example of a read that mapped to that region. Part of the read maps to the start of the duplication (dppA1, PA4496, indicated in red) and part to the end of the duplication (dppA3, PA4500, indicated in green). The center of the read maps to both genes. This region is where supposedly the target for the duplication through homologous recombination.
(TIFF)

S5 Fig Clones adapted through innovation are enriched in mutations in pre-existing duplicates. 10 cut-offs used. In gray: frequency of duplicates in the *P*. *aeruginosa* PAO1 genome; In green: frequency of duplicates in the set of mutated genes in clones that had to adapt through innovation; In orange: frequency of duplicates in the set of mutated genes in clones that had to adapt through optimization.
(TIF)

S6 Fig Clones that had to adapt through innovation have accumulated mutations with more negative pleiotropic effects. In gray: the number of effective doublings is not different from the ancestor. In green: the number of effective doublings is higher in the evolved clone than in the ancestor. In orange: the number of effective doublings is lower for the evolved clone than for the ancestor. X axis: assayed clones. Y axis: ancestor's growth: pale yellow: carbon sources where the number of viable cells per mL for the ancestor was lower than in the water well; orange: carbon sources where the ancestral strain grew poorly (number of viable cells per mL < 10^7.84^); red: carbon sources where the ancestral strain was able to grow (number of viable cells per mL > 10^7.84^).
(PDF)

S7 Fig Clustering analysis of viable cell titre (log10 cells/mL) and OD values for the ancestral PAO1 strain. The clustering analysis was done for the carbon sources where the experimental populations increased growth over time. Values are the average of 16 technical replicates per carbon source.
(TIFF)

S1 Table Effect of the mutations identified in clones that had to adapt through innovation and optimization. (DOC)

S2 Table List of mutations occurred in each carbon source and their putative adaptive role. Clones evolved through innovation have a higher frequency of mutations specific to the catabolism of the carbon source where they evolved than clones evolved through optimization. Clones evolved through optimization frequently have mutations that might be generally beneficial in laboratory conditions. Pathway information was obtained from the KEGG Database. Gene information was extracted from Pseudomonas Genome DB and Uniprot.
(DOC)

S3 Table COG classification of the mutated genes in clones that had to adapt through innovation and optimization. (DOC)

S4 Table Number of duplicates in the *P*. *aeruginosa* PAO1 genome and among the mutated genes in our experiment. We used 10 combinations of cut-offs of minimum length coverage and percentage of identical residues. In brackets there is the p-value for the Pearson's X^2^ test among the clones that had to evolve through innovation and optimization and PAO1 genome.
(DOC)

S5 Table Pleiotropic effects in clones that had to adapt through innovation and optimization. (DOC)

S6 Table Number of positive, average and negative cases of pleiotropic effects for clones carrying mutations in duplicated genes and clones carrying mutations in non-duplicated genes. (DOC)

S7 Table Viable cell titre (log10 cells/mL) and OD values for the ancestral strain in the carbon sources where the experimental populations increased growth over time. (DOC)

S8 Table Primers designed to confirm the genetic duplications in the clones evolved in Glycyl-L-Glutamic acid and Hydroxil-L-Proline. (DOC)
