# Supplementary figures and images for: The Genomic Basis of Evolutionary Innovation in Pseudomonas aeruginosa
Source: PLoS Genet. 2016 May 5;12(5):e1006005. doi: 10.1371/journal.pgen.1006005 (PMC4858143; doi:10.1371/journal.pgen.1006005)

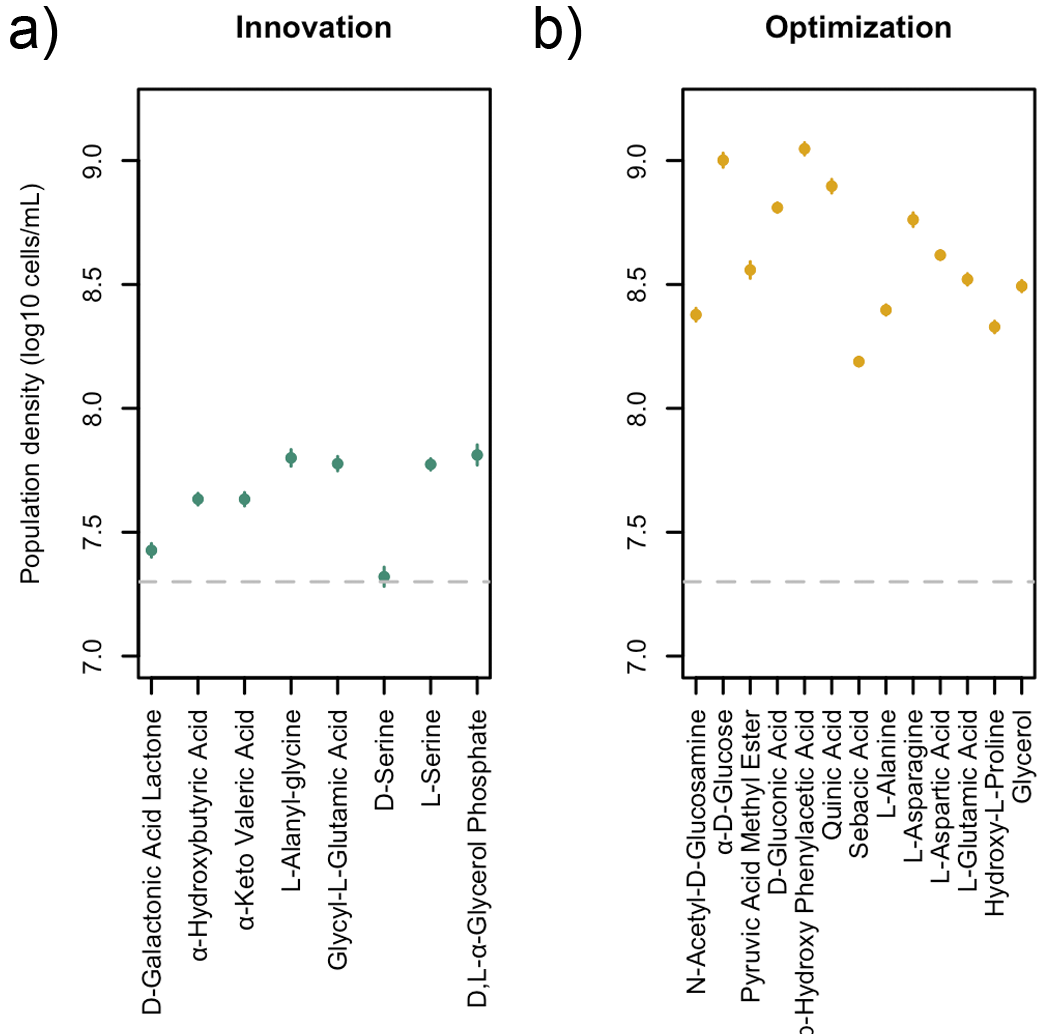

Supplement: S1 Fig — Panel a shows the substrates where the ancestral strain grew poorly (innovation) and panel b shows the substrates where the parental PAO1 was able to grow efficiently (optimization). Growth was calculated as viable cell titre (log10 cells/mL), 16 replicates per carbon source. (TIF) [file pgen.1006005.s002.tif]

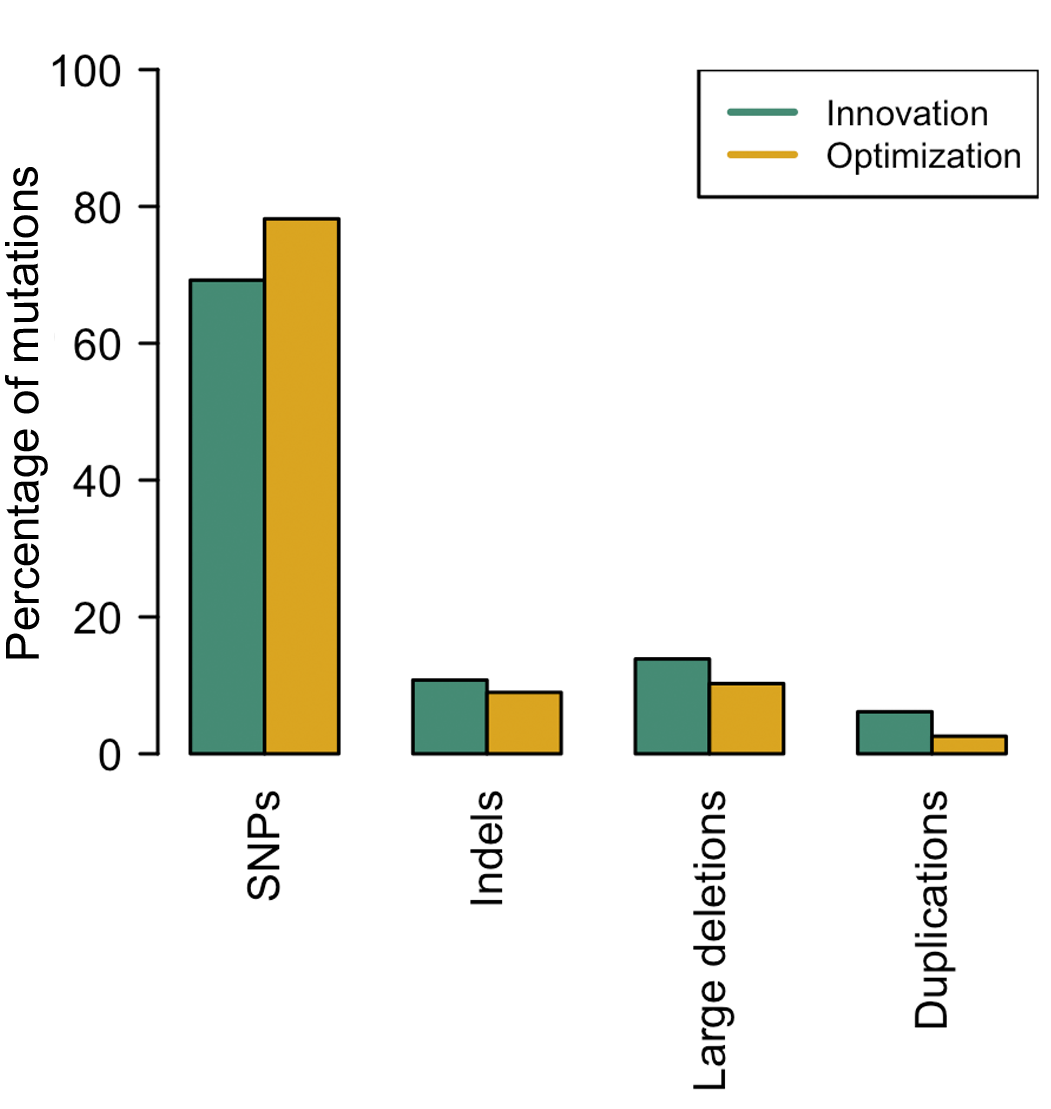

Supplement: S2 Fig — Large deletions > 30 bp. (TIF) [file pgen.1006005.s003.tif]

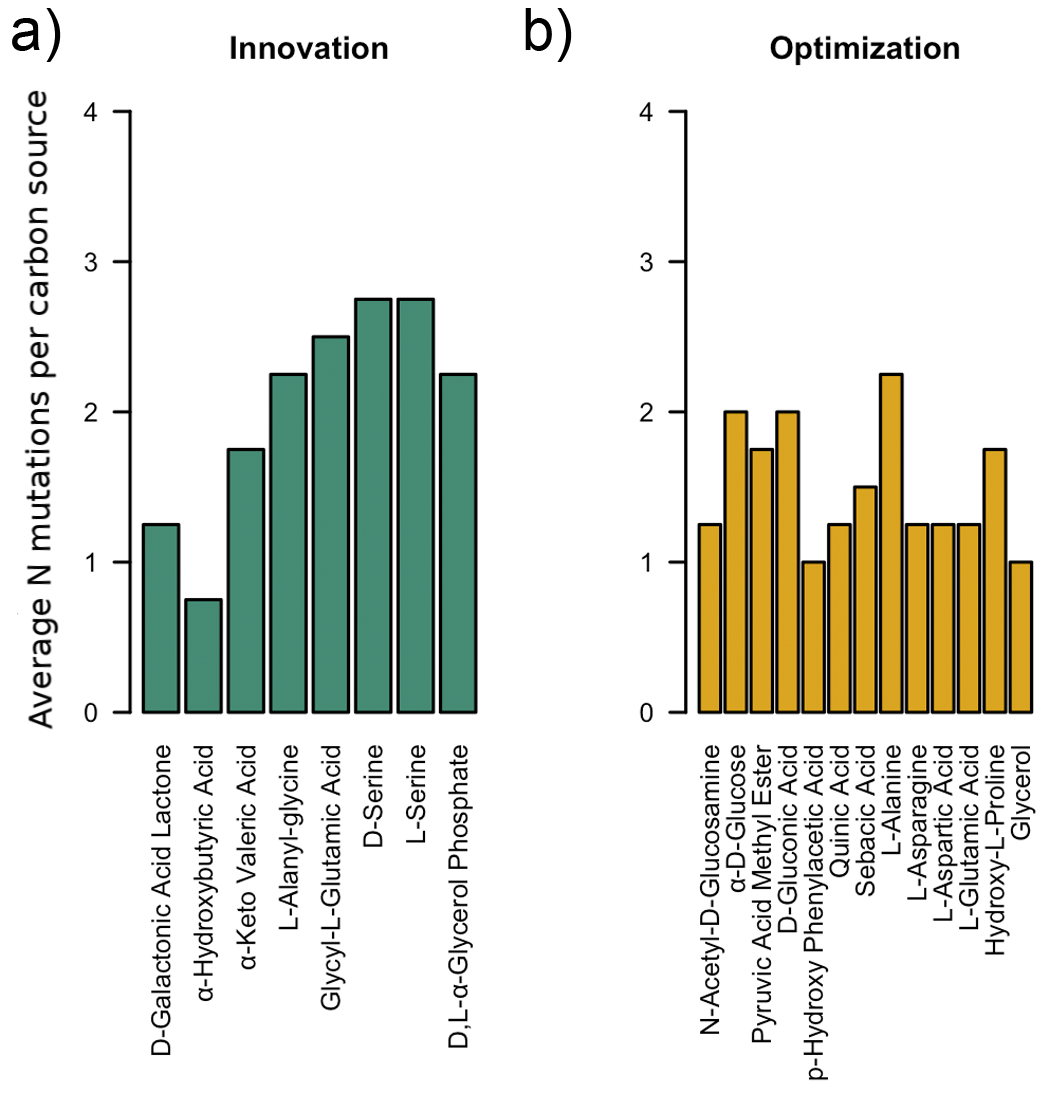

Supplement: S3 Fig — (TIF) [file pgen.1006005.s004.tif]

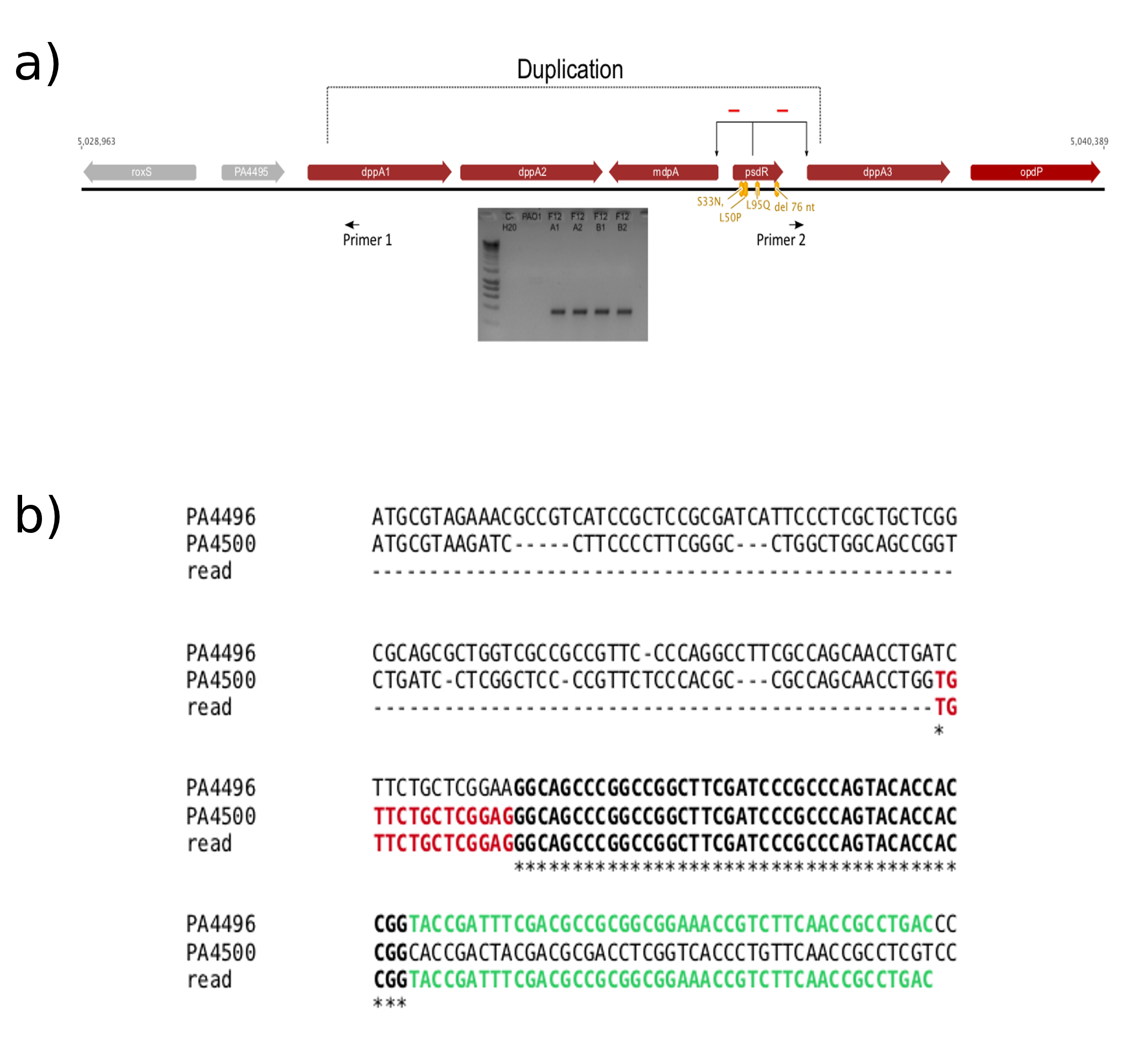

Supplement: S4 Fig — Panel a, The schematic diagram shows the genetic environment of the operon (red arrows) mutated in all the four clones that adapted to glycyl-L-glutamic acid. This operon is involved in di-peptide and amino acids transport. The reading frames for genes are shown as arrows, with the direction of transcription indicated by the arrowhead. PsdR is a transcriptional regulator that represses the expression of mdpA (metallopeptidase) and ddpA3 (dipeptide binding protein involved in the internalization of glycyl-L-glutamic acid), as indicated by the red minus symbols in the figure. Yellow ellipses indicate the positions of the mutations in psdR and the changes in the predicted protein sequence are described below them (“del 76 nt” indicates a deletion of 76 nucleotides). Dashed lines indicate the duplicated region between ddpA1 and ddpA3. The numbers in the left and right above the sequence indicate the genomic location in the P. aeruginosa PAO1 genome (NCBI taxonomy ID: 208964). We used Primer 1 (5’->3’,CGCGAGGCCGGGAAGGACCTT) and Primer 2 (5’->3’, TTGCCATGGCCCATAAGGCC) to confirm the duplications. The picture below the sequence shows the result of the agarose gel electrophoresis of the PCR products using primers 1 and 2. The first lane contains the molecular weight marker; the next two lanes are two negative controls using water and the DNA from the parental PAO1 strain as template for the PCR reaction (note that both reactions are negative). The remaining lanes show the result of the PCR using DNA samples from the four PAO1 clones tested in this experiment, which had evolved in the presence of glycyl-L-glutamic acid as the sole carbon source for 30 days (F12 A1, F12 A2, F12 B1, F12 B2). Note that the PCR reaction for all the clones is positive. We sequenced the PCR products confirming the results. Panel b, Tandem duplication as a result of homologous recombination. Nucleotide alignment between the 5' region of the genes dppA1(PA4496), dppA3 (PA4500) and an example of a re [file pgen.1006005.s005.tiff]

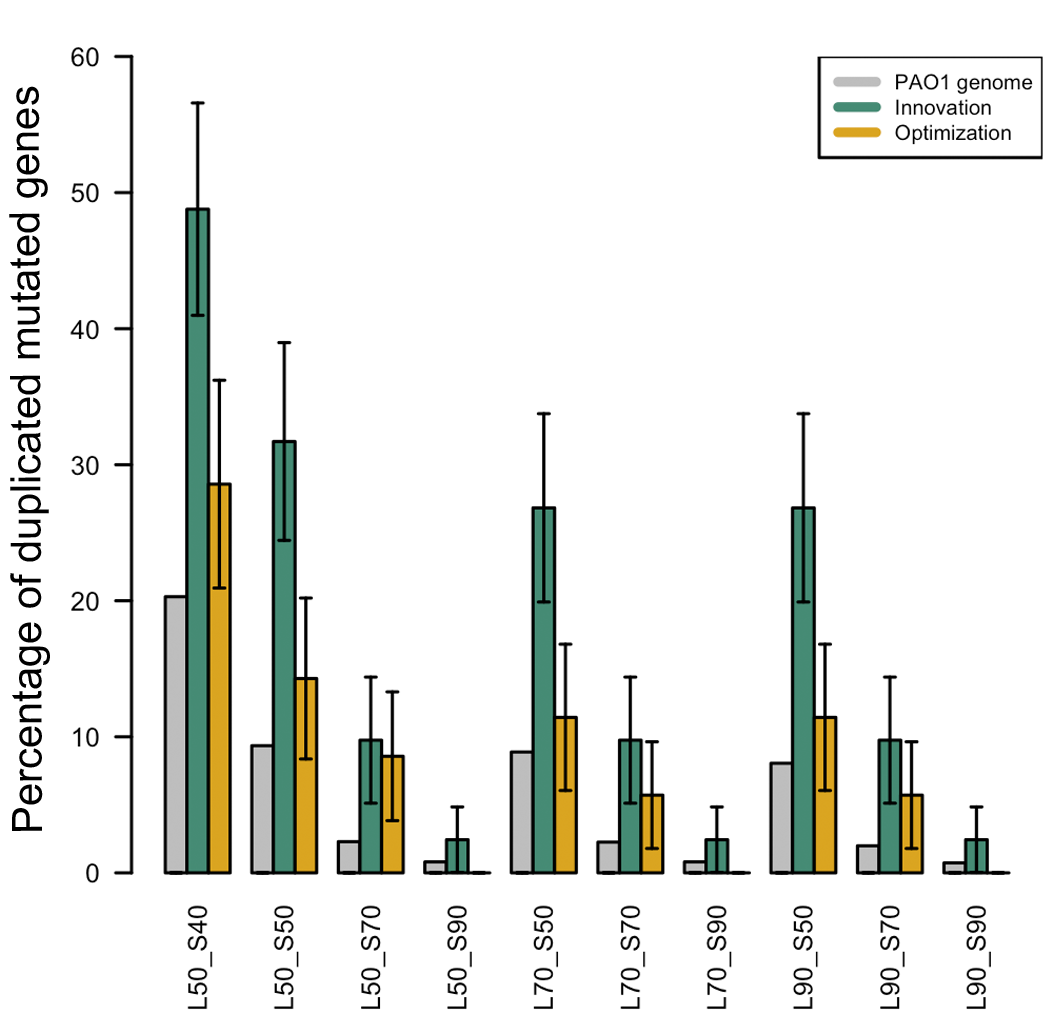

Supplement: S5 Fig — 10 cut-offs used. In gray: frequency of duplicates in the P. aeruginosa PAO1 genome; In green: frequency of duplicates in the set of mutated genes in clones that had to adapt through innovation; In orange: frequency of duplicates in the set of mutated genes in clones that had to adapt through optimization. (TIF) [file pgen.1006005.s006.tif]

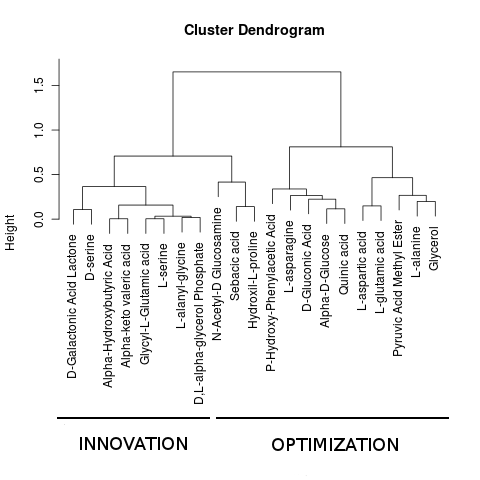

Supplement: S7 Fig — The clustering analysis was done for the carbon sources where the experimental populations increased growth over time. Values are the average of 16 technical replicates per carbon source. (TIFF) [file pgen.1006005.s008.tiff]
