# Supplementary material for: The Genomic Basis of Evolutionary Innovation in Pseudomonas aeruginosa
Source: PLoS Genet. 2016 May 5;12(5):e1006005. doi: 10.1371/journal.pgen.1006005 (PMC4858143; doi:10.1371/journal.pgen.1006005)
Supplement: S1 Table — (DOC) [file pgen.1006005.s009.doc]

**S1 Table.** Effect of the mutations identified in clones that had to adapt through innovation and optimization.

|  |  | **Innovation** |  | **Optimization** |  |
| --- | --- | --- | --- | --- | --- |
|  |  | N mutations | % | N mutations | % |
| **Point mutations** |  |  |  |  |  |
|  | Non-synonymous | 39 | 60 | 50 | 64.10 |
|  | Synonymous | 3 | 4.62 | 0 | 0 |
|  | Start codon lost | 0 | 0 | 1 | 1.28 |
|  | Stop codon gain | 2 | 3.08 | 5 | 6.41 |
|  | Intergenic | 1 | 1.54 | 5 | 6.41 |
| **Indels** |  |  |  |  |  |
|  | *Deletions* |  |  |  |  |
|  | Frameshift | 3 | 4.62 | 2 | 2.56 |
|  | Codon change + codon deletion | 2 | 3.08 | 2 | 2.56 |
|  | Codon deletion | 1 | 1.54 | 2 | 2.56 |
|  | *Insertions* |  |  |  |  |
|  | Frameshift | 1 | 1.54 | 0 | 0 |
|  | Intergenic | 0 | 0 | 1 | 1.28 |
| **Duplications** |  | 4 | 6.15 | 2 | 2.56 |
| **Large deletions** |  |  |  |  |  |
|  | Codon deletion | 1 | 1.54 | 1 | 1.28 |
|  | Codon deletion + intergenic | 1 | 1.54 | 0 | 0 |
|  | Codon change + codon deletion | 0 | 0 | 1 | 1.28 |
|  | Stop codon lost | 1 | 1.54 | 0 | 0 |
|  | Frameshift | 2 | 3.08 | 5 | 6.41 |
|  | Fusion 2 proteins | 1 (PA2444-PA2445) | 1.54 | 1 (PA1358-PA1359) | 1.28 |
|  | Intergenic | 3 | 4.62 | 0 | 0 |
