# Supplementary material for: The Genomic Basis of Evolutionary Innovation in Pseudomonas aeruginosa
Source: PLoS Genet. 2016 May 5;12(5):e1006005. doi: 10.1371/journal.pgen.1006005 (PMC4858143; doi:10.1371/journal.pgen.1006005)
Supplement: S2 Table — Clones evolved through innovation have a higher frequency of mutations specific to the catabolism of the carbon source where they evolved than clones evolved through optimization. Clones evolved through optimization frequently have mutations that might be generally beneficial in laboratory conditions. Pathway information was obtained from the KEGG Database. Gene information was extracted from Pseudomonas Genome DB and Uniprot. (DOC) [file pgen.1006005.s010.doc]

**S2 Table.** List of mutations occurred in each carbon source and their putative adaptive role. Clones evolved through innovation have a higher frequency of mutations specific to the catabolism of the carbon source where they evolved than clones evolved through optimization. Clones evolved through optimization frequently have mutations that might be generally beneficial in laboratory conditions. Pathway information was obtained from the KEGG Database. Gene information was extracted from Pseudomonas Genome DB and Uniprot. NS SNP: non-synonymous SNP, S SNP: synonymous SNP, nt: nucleotide.

| **Carbon source** | **Mutation** |
| --- | --- |
| **Innovation** |  |
| D-Galactonic Acid Lactone | All 4 clones have generally beneficial mutations (affecting motility, cell wall formation, respiratory chain). Mutated genes: PA1099 in clone B2, PA1104 in clone B2, PA2638 in clone A2, PA4954 in clone A1, PA5045 in clone A2. All mutations are NS SNPs except the mutation affecting PA5045, which is a SNP that introduces a premature stop codon and putatively truncates more than half of the protein. |
| α-Hydroxybutyric Acid | All 4 clones have generally beneficial mutations (affecting chemotaxis). Mutated genes: PA4309 in clone B1 (NS SNP), intergenic mutation at position 785623 in clone A1 and A2. This mutation deletes an intergenic region between a noncoding RNA (phrD) and the reverse transcripatase from the bacteriophage Pf1 (PA0715). The deleted region is an homopolymeric region (TCCCCGC)*18, and the deletion deletes 13 repetitions. This mutation introduces a frameshift that might affect the reverse transcripatse. |
| α-Keto Valeric Acid | 2 clones have mutations potentially specific to the carbon source where they evolved. PA2624 (NS SNP in clone A2) is an isocitrate dehydrogenase involved in Krebs cycle and PA3590 (S SNP in clone A1) is a 3-hydroxyacyl-CoA dehydrogenase that participates in ß-oxidation, which generates acetyl-CoA that can enter into the Krebs cycle. α-Keto Valeric Acid is an alpha-keto acid and alpha-keto acids are known to be involved in Krebs cycle and glycolysis. Clone A2 also has a 126 nucleotide deletion affecting an intergenic region. This mutation does not cause any frameshift. This mutation deletes the intergenic region between PA4937.1 and PA4937.2, which are two tRNA-Leu. Ketoacids are involved in the metabolism of valine, leucine and isoelucine. Therefore, this deletion might be adaptive.  3 clones have generally beneficial mutations (affecting transcriptional regulation and signal transduction). Mutated genes: PA4508 (NS SNP) in clone B1, PA4606 in clone A2 (31 nt deletion, which introduces a frameshift and putatively inactivates the protein) and B2 (NS SNP), PA5205 in clone B2 (NS SNP). |
| L-Alanyl-glycine | The 4 clones have mutations specific to the carbon source where they evolved. All clones have mutations in PA4499 (mutated in clone A1, A2, B1 and B2), which is a gene found in an operon involved in di-peptide and amino acid transport. This gene is the repressor of the operon [1]. All clones except one have NS SNP affecting PA4499. Clone A1 has 1 nt indel, which causes a frameshift.  All 4 clones have generally beneficial mutations (affecting transcriptional regulation and chemotaxis). Mutated genes: PA1003 in clone A2 (NS SNP), PA1561 in clone A2 (12 nt deletion), PA2867 in clone B1 (NS SNP), PA3002 in clone A1 (SNP causing the gain of a stop codon, which truncates around ¾ parts of the protein), PA3348 in clone B2 (NS SNP). |
| Glycyl-L-Glutamic Acid | The 4 clones have mutations specific to the carbon source where they evolved. The 4 clones (A1, A2, B1 and B2) have two types of mutations affecting an operon involved in di-peptide and amino acid transport. First, they have mutations potentially inactivating the repressor of the operon (PA4499). 3 clones have NS SNPs (clones A1, B1, B2) and 1 clone (A2) has a deletion of 76 nt, which deletes the C-terminal part of the protein. Secondly, they have a tandem duplication affecting the genes PA4496-PA4500 [1]. Additionally, clone B1 also has a 70 nt deletion in gene PA4500, which causes a frameshift.  1 clone has generally beneficial mutations (affecting chemotaxis). Mutated gene: PA1561 in clone A2. |
| D-Serine | The 4 clones have mutations specific to the carbon source where they evolved. PA0789 (12 nt deletion in clone B1 and NS SNP in B2) is an amino acid permease, PA0904 (NS SNP in clone B2) is an aspartate kinase involved in amino acid metabolism, PA1971 (NS SNP in clone A2) is a branched chain amino acid transporter, PA2897 (NS SNP in clone A1) is an aminotransferase, PA5304 (NS SNP in clone A1) is a D-amino acid dehydrogenase. Clone A2 also has a SNP in the intergenic region upstream the gene D-amino acid dehydrogenase (PA5304), putatively affecting its promoter.  3 clones have generally beneficial mutations (affecting motility and transcriptional regulation). Mutated genes: PA1099 in clone A2 (NS SNP), PA1447 in clone A1 (21 nt deletion), PA1448 in clone B1 (2 nt insertion, causing a frameshift), PA1581 in clone B1 (NS SNP). |
| L-Serine | The 4 clones have mutations specific to the carbon source where they evolved. The 4 clones have NS SNP affecting the gene PA2449, clones B1 and B2 have the exact same mutation. PA2449 is a gene known to be involved in serine metabolism [2]. All clones have additional mutations potentially affecting L-Serine metabolism. Clone A1 has a NS SNP in PA3569 (3-hydroxyisobutyrate dehydrogenase), which is an oxidoreductase that has L-Serine as a substrate [3]. Clone B1 has a deletion that affects the N-terminal part of PA2444 and the C-terminal part of PA2445, these are genes involved in serine metabolism [2]. Clone A2 has a NS SNP in PA0789, which is an amino acid permease. Clone A1 has a NS SNP in PA4695, an acetolactate synthase involved in the biosynthesis of valine, leucine and isoleucine. Serine is the precursor of several amino acids, such as valine, leucine and isoleucine.  3 clones have generally beneficial mutations (affecting chemotaxis). Mutated genes: PA1561 (NS SNP) in clone A2, PA4208 (S SNP) in clones B1 and B2. |
| D,L-α-Glycerol Phosphate | The 4 clones have mutations specific to the carbon source where they evolved. 3 clones have specific mutations affecting betaine metabolism (PA5373 in clone A2, PA5374 in clone A2 and B2, PA5380 in clone B1). D,L-α-Glycerol Phosphate (or sn-glycerol 3-phosphate) is a glycerophospholipid and one of the products of glycerophospholipid metabolism is choline. Choline can be oxidized to obtain glycine betaine, which can be utilized as a sole carbon source by *P. aeruginosa*. These clones have putatively inactivating mutations (frameshifts and deletions) in two proteins involved in betaine metabolism, PA5374 (transcriptional regulator BetI) which represses the synthesis of glycine betaine, and PA5380 (glycine betaine- and dimethylglycine-responsive regulator), which induces the genes involved in glycine betaine catabolism [4]. Therefore, these mutations putatively increase the availability of glycine betaine. All 4 clones also have NS SNPs in RNA polymerase-binding transcription factor DksA (PA4723, mutated in clones A1, A2, B1 and B2). *DksA* potentiates the regulation of rRNA promoters by guanosine 5′-diphosphate 3′-diphosphate (ppGpp) [5] and ppGpp regulates *PlsB* activity [6]. *PlsB* is a glycerol-3-phosphate acyltransferase that catalyzes the following reaction: Acyl-CoA + sn-glycerol 3-phosphate = CoA + 1-acyl-sn-glycerol 3-phosphate. High levels of ppGpp inhibit *PlsB* [7]. Finally, clone A1 has an additional NS SNP in RNA polymerase sigma factor RpoS (PA3622). This is the only clone that does not have mutations affecting betaine metabolism. |
| **Optimization** |  |
| N-Acetyl-D-Glucosamine | 2 clones have mutations specific to the carbon source where they evolved. PA0337 (NS SNP in clone A1) is a phosphoenolpyruvate-protein phosphotransferase that is member of the phosphotransferase system which transports sugars. N-Acetyl-D-Glucosamine is a monosaccharide. PA1304 (NS SNP in clone B1) is an oligopeptidase.  2 clones have generally beneficial mutations (affecting chemotaxis, two-component regulatory system and motility). Mutated genes: PA1452 in clone A1 (stop codon gained), PA1561 in clone B2 (NS SNP), PA3271 in clone B2 (NS SNP). |
| α-D-Glucose | All 4 clones have generally beneficial mutations (affecting chemotaxis). Mutated genes: PA1561 in clone A1 and B1 (NS SNP), PA2867 in clone B2 (NS SNP), PA3348 in clones A1, A2 and B1 (NS SNP), PA5017 in clone B2 (NS SNP). |
| Pyruvic Acid Methyl Ester | All 4 clones have generally beneficial mutations (affecting chemotaxis and two-component regulatory system). Mutated genes: PA1561 in clone A1 (in frame codon deletion) and A2 (NS SNP), PA3271 in clones A2 (stop codon gained), B1 (NS SNP) and B2 (NS SNP), PA3348 in clone B2 (NS SNP). |
| D-Gluconic Acid | 2 clones have mutations specific to the carbon source where they evolved. Gluconic acid is a compound originated from the oxidation of glucose. PA3924 (NS SNP in clone A2) is a long-chain-fatty-acid--CoA ligase involved in lipid metabolism. PA4127 (codon change + codon deletion in clone A1) is a 2-oxo-hepta-3-ene-1,7-dioic acid hydratase involved in the degradation of 4-hydroxyphenylacetate to obtain pyruvate and succinate semialdehyde.  All 4 clones have generally beneficial mutations (affecting chemotaxis and biofilm formation). Mutated genes: PA1561 in clones B1 and B2 (NS SNPs), PA2231 in clone A2 (NS SNP), PA2867 in clone B2 (NS SNP), PA3349 in clone A1 (NS SNP). Clone A2 has a SNP affecting an intergenic region. The SNP is downstream gene *himD* (integration host factor beta subunit, PA3161) and upsteram the gene *wzz* (O-antigen chain length regulator, PA3160). |
| P-Hydroxy Phenylacetic Acid | All 4 clones have generally beneficial mutations (affecting chemotaxis). Mutated genes: PA1458 in clones A1 and A2 (NS SNP), PA4309 in clone B1 (codon change+codon deletion), PA5017 in clone B2 (stop codon gained). |
| Quinic Acid | All 4 clones have generally beneficial mutations (affecting chemotaxis and transcriptional regulation). Mutated genes: PA1003 in clone A1 (codon deletion), PA4309 in clones A2 and B2 (NS SNP), PA4310 in clones A1 and B1 (NS SNP). |
| Sebacic Acid | 2 clones have mutations specific to the carbon source where they evolved. Sebacic acid is a dicarboxylic acid. PA2550 (NS SNPs in clones B1 and B2) is an acyl-CoA dehydrogenase involved in fatty acid metabolism.  2 clones have generally beneficial mutations (motility, cell wall and transcriptional regulation). Mutated genes: PA1015 in clone A2 (NS SNP), PA1097 in clone B1 (NS SNP), PA5045 in clone A1 and A2 (stop codon gained). |
| L-Alanine | 3 clones have mutations specific to the carbon source where they evolved. They have NS SNPs in the histidine utilization (hut) operon, which is involved in the pathway to degrade L-histidine into L-glutamate [8]. PA5097 (*hutT*) (mutated in clone B2) is an amino acid permease. PA5100 (*hutU*) (mutated in clones B1 and B2) is an urocanate hydratase. PA5105 (*hutC*) (mutated in clone A1) is the repressor of the *hut* operon. And L-glutamate is involved in L-Alanine metabolism.  All 4 clones have generally beneficial mutations (affecting transcriptional regulation, chemotaxis and biofilm formation). Mutated genes: PA2234 in clone A1 (1 nt deletion, introducing a frameshift which putatively inactivates the protein), PA2235 in clone A2 (codon deletion), PA3711 in clones A2 and B1 (NS SNP), PA4309 in clone B2 (NS SNP). |
| L-Asparagine | All 4 clones have generally beneficial mutations (affecting chemotaxis). Mutated genes: PA1561 in clones A1 (NS SNP), B1 and B2 (169 nt deletion that introduces a frameshift), PA2228 in clone A1, PA2229 in clones A1 and A2. Clone A1 has a deletion of 2712 nt that partially deletes PA2228 and completely deletes PA2229. Clone A2 has a large deletion of 121669 nt, which affects the genes PA2229-PA2332. Those genes are related with quorum sensing. |
| L-Aspartic Acid | 3 clones have mutations specific to the carbon source where they evolved. PA1336 (NS SNP in clones A1 and B1) is an histidine kinase, and is member of the *aau* system, which has been shown to metabolize aspartate and glutamate in *Pseudomonas putida* [9]. There is also an intergenic mutation in the promoter region of PA5479 (in clone B1), which is a proton-glutamate symporter. In *Bacillus subtilis* this gene is a transporter specific for L-glutamate and L-aspartate [10].  1 clone has generally beneficial mutations (affecting transcriptional regulation and chemotaxis). Mutated genes: PA1003 in clone A2 (775 nt deletion, which introduces a frameshift), PA1561 in clone A2 (start codon lost). |
| L-Glutamic Acid | The 4 clones have mutations specific to the carbon source where they evolved. PA1335 (NS SNP in clone B2) is a two-component regulator, PA1336 (NS SNP in clone A2) is an histidine kinase. Both genes form the system *aau* which metabolizes aspartate and glutamate [9]. There is also an intergenic mutation in the promoter region of PA5479 (mutated in clone A1 and B1), which is a proton-glutamate symporter. In *Bacillus subtilis* this gene is a transporter specific for L-glutamate and L-aspartate [10].  1 clone has a generally beneficial mutation (affecting chemotaxis). Mutated gene: PA2867 in clone A1 (NS SNP). |
| Hydroxy-L-Proline | 1 clone has a mutation specific to the carbon source where it evolved. PA0903 (NS SNP in clone B1) is a alanyl-tRNA synthetase  ATP + L-alanine + tRNA(Ala) = AMP + diphosphate + L-alanyl-tRNA(Ala).  3 clones have generally beneficial mutations (large gene deletions, chemotaxis and respiratory chain). Mutated genes: PA1359 in clone B2 (NS SNP), PA1561 in clone A1 (NS SNP), PA1358 in clone A2, PA1359 in clone A2, large duplications in clones A1 and B1 affecting more than 350000 nt. Clone A2 has a deletion of 172 nt that deletes partially both PA1358 and PA1359 and introduces a frameshift in both genes. Clone B2 has 1 nucleotide insertion in a region upstream a pseudogene (PA4641) that contains a frameshift. The insertion affects a homopolymeric tract containing 8C and adds an extra C. This insertion does not seem to create a new ORF. |
| Glycerol | The 4 clones have mutations specific to the carbon source where they evolved. Clone B1 has an intergenic mutation just 5 nt before the start of a probable glyceraldehyde-3-phosphate dehydrogenase (PA2323). The remaining 3 clones have specific mutations affecting the coding region or the promoter of PA2320 (mutated in clones A1, A2, and B2). Clone A1 has a NS SNP, clone A2 a 11 nt deletion that introduces a frameshift and clone B2 has an in-frame deletion of 354 nt. PA2320 is the transcriptional regulator GntR that is found in the gluconate operon. GntR represses the gluconate operon and also *edd* (6-phosphogluconate dehydratase) and *eda* (2-keto-3-deoxy-6-phosphogluconate aldolase) genes [11]. *Edd* and *eda* are two enzymes of the Entner-Doudoroff pathway, which is used to metabolize carbohydrates and happens to be cyclic in *P. aeruginosa* [12]. *Edd* and *eda* are induced when *P. aeruginosa* is grown on glycerol [12]. Glycerol metabolism is done through its conversion to D-glyceraldehyde-3-phosphate, which can be recycled through the Entner-Doudoroff cycle [12]. The mutations observed in all 4 clones putatively inactivate GntR, therefore suppressing its negative regulation and promoting the Entner-Doudoroff pathway. |

Pathway information was obtained from the KEGG Database [13]. Gene information was extracted from Pseudomonas Genome DB [14] and Uniprot [15].

**References**

1. Kiely PD, O’Callaghan J, Abbas A, O’Gara F. Genetic analysis of genes involved in dipeptide metabolism and cytotoxicity in Pseudomonas aeruginosa PAO1. Microbiology. 2008; 154: 2209–18.

2. Lundgren BR, et al. Gene PA2449 is essential for glycine metabolism and pyocyanin biosynthesis in Pseudomonas aeruginosa PAO1. J. Bacteriol. 2013; 195: 2087–100.

3. Chowdhury EK, Nagata S, Misono H. 3-Hydroxyisobutyrate dehydrogenase from Pseudomonas putida E23: purification and characterization. Biosci. Biotechnol. Biochem. 1996; 60: 2043–7.

4. Wargo MJ, Szwergold BS, Hogan DA. Identification of two gene clusters and a transcriptional regulator required for Pseudomonas aeruginosa glycine betaine catabolism. J. Bacteriol. 2008; 190: 2690–9.

5. Paul BJ, et al. DksA: a critical component of the transcription initiation machinery that potentiates the regulation of rRNA promoters by ppGpp and the initiating NTP. Cell. 2004; 118: 311–22.

6. Magnusson LU, Farewell A, Nyström T. ppGpp: a global regulator in Escherichia coli. Trends Microbiol. 2005; 13(5): 236–42.

7. Heath RJ, Jackowski S, Rock CO. Guanosine tetraphosphate inhibition of fatty acid and phospholipid synthesis in Escherichia coli is relieved by overexpression of glycerol-3-phosphate acyltransferase (plsB). J. Biol. Chem. 1994; 269: 26584–90.

8. Zhang XX, Rainey PB. Genetic analysis of the histidine utilization (hut) genes in Pseudomonas fluorescens SBW25. Genetics. 2007; 176: 2165–76.

9. Sonawane AM, Singh B, Röhm KH. The AauR-AauS two-component system regulates uptake and metabolism of acidic amino acids in Pseudomonas putida. Appl. Environ. Microbiol. 2006; 72: 6569–77.

10. Tolner B, Ubbink-Kok T, Poolman B, Konings WN. Characterization of the proton/glutamate symport protein of Bacillus subtilis and its functional expression in Escherichia coli. J. Bacteriol. 1995; 177: 2863–9.

11. Tong S, Porco A, Isturiz T, Conway T. Cloning and molecular genetic characterization of the Escherichia coli gntR, gntK, and gntU genes of GntI, the main system for gluconate metabolism. J. Bacteriol. 1996; 178: 3260–9.

12. Lessie TG, Phibbs PV. Alternative pathways of carbohydrate utilization in pseudomonads. Annu. Rev. Microbiol. 1984; 38: 359–88.

13. Kanehisa M, et al. Data, information, knowledge and principle: back to metabolism in KEGG. Nucleic Acids Res. 2014; 42: D199–205.

14. Winsor GL, et al. Pseudomonas Genome Database: improved comparative analysis and population genomics capability for Pseudomonas genomes. Nucleic Acids Res. 2011; 39: D596–600.

15. UniProt Consortium. UniProt: a hub for protein information. Nucleic Acids Res. 2014; 43: D204–12.
