# Supplementary material for: The Genomic Basis of Evolutionary Innovation in Pseudomonas aeruginosa
Source: PLoS Genet. 2016 May 5;12(5):e1006005. doi: 10.1371/journal.pgen.1006005 (PMC4858143; doi:10.1371/journal.pgen.1006005)
Supplement: S3 Table — (DOC) [file pgen.1006005.s011.doc]

**S3 Table.** COG classification of the mutated genes in clones that had to adapt through innovation and optimization.

| COG | Innovation | Optimization | N in the PAO1 genome* |
| --- | --- | --- | --- |
| **Cellular process and signaling** |  |  |  |
| Signal transduction mechanisms (T) | 8 (9.52%) | 8 (11.27%) | 176 (3.72%) |
| Cell motility (N) & Intracellular trafficking, secretion and vesicular transport (U) | 3 (3.57%) | 1 (1.41%) | 76 (1.61%) |
| Cell motility (N) & Signal transduction mechanisms (T) | 6 (7.14%) | 29 (40.85%) | 47 (0.99%) |
| Cell wall/membrane/envelope biogenesis (M) & Intracellular trafficking, secretion and vesicular transport (U) | 2 (2.38%) | 0 | 19 (0.40%) |
| Cell motility (N) | 1 (1.19%) | 0 | 28 (0.59%) |
| Cell wall/membrane/envelope biogenesis/Intracellular trafficking, secretion and vesicular transport (M) | 1 (1.19%) | 5 (7.04%) | 223 (4.71%) |
| Defense mechanisms (V) | 0 | 1 (1.41%) | 78 (1.65%) |
| Intracellular trafficking, secretion and vesicular transport (U) | 1 (1.19%) | 0 | 69 (1.46%) |
| **Metabolism** |  |  |  |
| Amino acid transport and metabolism (E) | 31 (36.90%) | 9 (12.68%) | 398 (8.41%) |
| Energy production and conversion (C) | 4 (4.76%) | 0 | 292 (6.17%) |
| Lipid transport and metabolism (I) | 2 (2.38%) | 2 (2.82%) | 180 (3.80%) |
| Secondary metabolites biosynthesis, transport and catabolism (Q) | 0 | 1 (1.41%) | 100 (2.11%) |
| Lipid transport and metabolism (I) & Secondary metabolites biosynthesis, transport and catabolism (Q) | 0 | 1 (1.41%) | 50 (1.06%) |
| **Transcription (K)** | 18 (21.43%) | 11 (15.49%) | 388 (8.20%) |
| **Transcription & Metabolism** |  |  |  |
| Transcription (K) & Amino acid transport and metabolism (E) | 5 (5.95%) | 0 | 11 (0.23%) |
| **Others** |  |  |  |
| Replication, recombination and repair (L) & Transcription (K) | 1 (1.19%) | 0 | 8 (0.17%) |
| Translation, ribosomal structure and biogenesis (J) | 0 | 1 (1.41%) | 189 (3.99%) |
| Function unknown (S) | 1 (1.19%) | 2 (2.82%) | 512 (10.82%) |

COG categories were counted by mutation, i.e. if a gene was mutated twice in the dataset, it's COG category was counted twice.

^a^ Number of genes in the PAO1 genome annotated with this COG category. In brackets frequency of genes with a specific COG category, out of the total number of genes with an assigned COG category (4731)
