# Supplementary material for: The Genomic Basis of Evolutionary Innovation in Pseudomonas aeruginosa
Source: PLoS Genet. 2016 May 5;12(5):e1006005. doi: 10.1371/journal.pgen.1006005 (PMC4858143; doi:10.1371/journal.pgen.1006005)
Supplement: S4 Table — We used 10 combinations of cut-offs of minimum length coverage and percentage of identical residues. In brackets there is the p-value for the Pearson's X2 test among the clones that had to evolve through innovation and optimization and PAO1 genome. (DOC) [file pgen.1006005.s012.doc]

**S4 Table.** Number of duplicates in the PAO1 genome and among the mutated genes in our experiment. We used 10 combinations of cut-offs of minimum length coverage and percentage of identical residues. In brackets there is the p-value for the Pearson's X^2^ test among the clones that had to evolve through innovation and optimization and PAO1 genome.

| **BlastClust cut-off** | **N duplicates**  **PAO1 genome** | **N duplicates in clones adapted through innovation** | **N duplicates in clones adapted through optimization** |
| --- | --- | --- | --- |
| 90 coverage, 90 identity | 41 (0.7%) | 1 (2.4%) (p-value = 0.7253) | 0 (NA) |
| 90 coverage, 70 identity | 111 (2%) | 4 (9.7%) (p-value =0.003248*****) | 2 (5.7%) (p-value = 0.3376) |
| 90 coverage, 50 identity | 449 (8.1%) | 11 (26.8%) (p-value = 4.5e-05*****) | 4 (11.4%) (p-value = 0.6757) |
| 70 coverage, 90 identity | 45 (0.8%) | 1 (2.4%) (p-value = 0.7755) | 0 (NA) |
| 70 coverage, 70 identity | 126 (2.3%) | 4 (11.4%) (p-value = 0.007865*****) | 2 (5.7%) (p-value = 0.4261) |
| 70 coverage, 50 identity | 495 (8.9%) | 11 (26.8%) (p-value= 0.0001962*****) | 4 (11.4%) (p-value= 0.8186) |
| 50 coverage, 90 identity | 45 (0.8%) | 1 (2.4%) (p-value = 0.7755) | 0 (NA) |
| 50 coverage, 70 identity | 128 (2.3%) | 4 (9.8%) (p-value = 0.008716*****) | 3 (8.6%) (p-value = 0.05898) |
| 50 coverage, 50 identity | 521 (9.35%) | 13 (31.7%) (p-value = 4.345e-06*****) | 5 (14.3%) (p-value = 0.4792) |
| 50 coverage, 40 identity | 1131 (20.3%) | 20 (48.8%) (p-value= 1.659e-05*****) | 10 (28,6%) (p-value= 0.3166) |
| Total N genes | 5572 | 41 | 35 |
