# Supplementary material for: The Genomic Basis of Evolutionary Innovation in Pseudomonas aeruginosa
Source: PLoS Genet. 2016 May 5;12(5):e1006005. doi: 10.1371/journal.pgen.1006005 (PMC4858143; doi:10.1371/journal.pgen.1006005)
Supplement: S5 Table — (DOC) [file pgen.1006005.s013.doc]

**S5 Table.** Pleiotropic effects in clones that had to adapt through innovation and optimization.

| **Type strain** | **Positive effect** | **Neutral effect** | **Negative effect** |
| --- | --- | --- | --- |
| **Innovation (16 clones)** |  |  |  |
| Total number of pleiotropic effects | 263 | 924 | 317 |
| Total/ N clones | 16.44 | 57.75 | 19.81 |
| **Optimization (26 clones)** |  |  |  |
| Total number of pleiotropic effects | 443 | 1698 | 303 |
| Total/ N clones | 17.04 | 65.31 | 11.65 |

The total number of pleiotropic effects is the sum of pleiotropic effects (positive, neutral or negative) for all assayed clones for each category, innovation or optimization.
