# Supplementary material for: The Genomic Basis of Evolutionary Innovation in Pseudomonas aeruginosa
Source: PLoS Genet. 2016 May 5;12(5):e1006005. doi: 10.1371/journal.pgen.1006005 (PMC4858143; doi:10.1371/journal.pgen.1006005)
Supplement: S6 Table — (DOC) [file pgen.1006005.s014.doc]

**S6 Table.** Number of positive, average and negative cases of pleiotropic effects for clones carrying mutations in duplicated genes and clones carrying mutations in non-duplicated genes.

|  | N clones | Number cases of  + pleiotropy | Number cases of  - pleiotropy | Number cases of average pleiotropy |
| --- | --- | --- | --- | --- |
| **Recent duplicates** |  |  |  |  |
| Clones carrying mutations in recent duplicates | 9 | 200 | 88 | 558 |
| Clones not carrying mutations in recent duplicates | 33 | 506 | 532 | 2064 |
| **Ancient duplicates** |  |  |  |  |
| Clones carrying mutations in ancient duplicates | 13 | 138 | 301 | 783 |
| Clones not carrying mutations in ancient duplicates | 25 | 441 | 310 | 1599 |
| **All duplicates** |  |  |  |  |
| Clones carrying mutations in ancient and recent duplicates | 22 | 338 | 389 | 1341 |
| Clones not carrying mutations in duplicates | 20 | 368 | 231 | 1281 |
