# Supplementary material for: The Genomic Basis of Evolutionary Innovation in Pseudomonas aeruginosa
Source: PLoS Genet. 2016 May 5;12(5):e1006005. doi: 10.1371/journal.pgen.1006005 (PMC4858143; doi:10.1371/journal.pgen.1006005)
Supplement: S7 Table — (DOC) [file pgen.1006005.s015.doc]

**S7 Table.** Viable cell titre (log10 cells/mL) and OD values for the ancestral strain in the carbon sources where the experimental populations increased growth over time.

| **Carbon source** | **Viable cell titre** | **Std dev Viable cell titre** | **Prob. Innovation** | **Prob. Optimization** | **OD** | **Std dev OD** |
| --- | --- | --- | --- | --- | --- | --- |
| **Innovation** |  |  |  |  |  |  |
| D-Galactonic Acid Lactone | 7.427 | 0.028 | 0.990 | 0.010 | 0.005 | 0.003 |
| α-Hydroxybutyric Acid | 7.633 | 0.024 | 0.934 | 0.066 | 0.010 | 0.002 |
| α-Keto Valeric Acid | 7.633 | 0.027 | 0.934 | 0.066 | 0.014 | 0.002 |
| L-Alanyl-glycine | 7.800 | 0.034 | 0.639 | 0.361 | 0.015 | 0.005 |
| Glycyl-L-Glutamic Acid | 7.777 | 0.029 | 0.703 | 0.297 | 0.009 | 0.003 |
| D-Serine | 7.320 | 0.039 | 0.995 | 0.005 | -0.002 | 0.005 |
| L-Serine | 7.774 | 0.023 | 0.711 | 0.289 | 0.012 | 0.003 |
| D,L-α-Glycerol Phosphate | 7.812 | 0.041 | 0.596 | 0.404 | 0.030 | 0.064 |
| **Optimization** |  |  |  |  |  |  |
| N-acetyl-D Glucosamine | 8.378 | 0.026 | 3.145e-05 | 1.00 | 0.501 | 0.198 |
| α-D-Glucose | 9.001 | 0.029 | 3.731e-13 | 1.00 | 1.761 | 0.151 |
| Pyruvic Acid Methyl Ester | 8.559 | 0.034 | 3.211e-07 | 1.00 | 1.065 | 0.208 |
| D-Gluconic Acid | 8.810 | 0.019 | 2.053e-10 | 1.00 | 1.903 | 0.068 |
| P-Hydroxy Phenylacetic Acid | 9.048 | 0.025 | 9.308e-14 | 1.00 | 2.008 | 0.118 |
| Quinic Acid | 8.897 | 0.028 | 1.292e-11 | 1.00 | 1.711 | 0.062 |
| Sebacic Acid | 8.189 | 0.018 | 0.002 | 0.998 | 0.118 | 0.031 |
| L-Alanine | 8.397 | 0.021 | 1.877e-05 | 1.00 | 0.925 | 0.124 |
| L-Asparagine | 8.762 | 0.028 | 9.259e-10 | 1.00 | 1.587 | 0.150 |
| L-Aspartic Acid | 8.619 | 0.018 | 6.143e-08 | 1.00 | 1.306 | 0.092 |
| L-Glutamic Acid | 8.521 | 0.023 | 8.6703^-07^ | 1.00 | 1.418 | 0.109 |
| Hydroxy-L-Proline | 8.328 | 0.024 | 0.000 | 1.00 | 0.099 | 0.027 |
| Glycerol | 8.493 | 0.023 | 1.744e-06 | 1.00 | 0.751 | 0.098 |

Population density and OD are the average values obtained from 16 technical replicates. Probability of Innovation and Optimization computed with the mixtool package in R. OD values are blank corrected. Population density is measured as log10 cells/mL.
