# Supplementary material for: The Genomic Basis of Evolutionary Innovation in Pseudomonas aeruginosa
Source: PLoS Genet. 2016 May 5;12(5):e1006005. doi: 10.1371/journal.pgen.1006005 (PMC4858143; doi:10.1371/journal.pgen.1006005)
Supplement: S8 Table — (DOC) [file pgen.1006005.s016.doc]

**S8 Table.** Primers designed to confirm the genetic duplications in the clones evolved in Glycyl-L-Glutamic acid and Hydroxil-L-Proline.

| **Name** | **Target gene** | **Sequence 5’->3’** |
| --- | --- | --- |
| GLG-Fw | PA4499-PA4500 (intergenic) | TTGCCATGGCCCATAAGGCC |
| GLG-Rv | PA4496 | CGCGAGGCCGGGAAGGACCTT |
| HLP-Fw1 | PA1418 | ACCGCCAGGCTGTAGTAGA |
| HLP-Rv1 | PA1101 | TTGAGGCTGCCATAGAGCG |
| HLP-Fw2 | PA1609 | TTCCGGCGTCTCGTTGTAC |
| HLP-Rv2 | PA1156-PA1157 (intergenic) | ATCTTGGGTTTCGAGCGCA |

PCRs were performed using GoTaq green Mastermix (Promega, USA) and DNA samples from all the evolved clones in each environment. We use water and DNA from the parental strain PAO1 as negative controls. The amplification products were sequenced to confirm the results.
