# Supplementary material for: The Genomic Basis of Evolutionary Innovation in Pseudomonas aeruginosa
Source: PLoS Genet. 2016 May 5;12(5):e1006005. doi: 10.1371/journal.pgen.1006005 (PMC4858143; doi:10.1371/journal.pgen.1006005)
Supplement: S6 Fig — In gray: the number of effective doublings is not different from the ancestor. In green: the number of effective doublings is higher in the evolved clone than in the ancestor. In orange: the number of effective doublings is lower for the evolved clone than for the ancestor. X axis: assayed clones. Y axis: ancestor's growth: pale yellow: carbon sources where the number of viable cells per mL for the ancestor was lower than in the water well; orange: carbon sources where the ancestral strain grew poorly (number of viable cells per mL < 107.84); red: carbon sources where the ancestral strain was able to grow (number of viable cells per mL > 107.84). (PDF) [file pgen.1006005.s007.pdf]

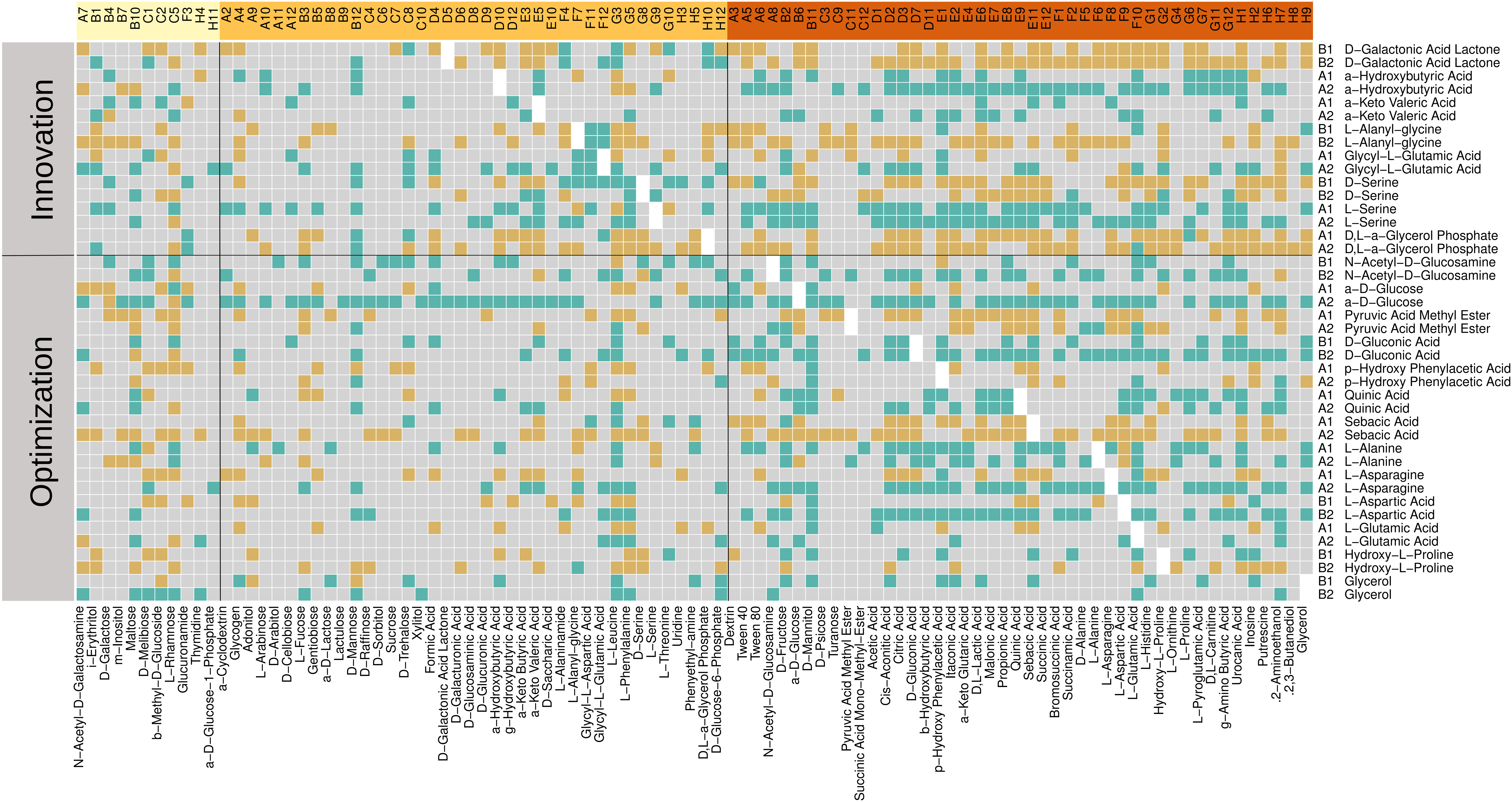
Innovation
Optimization
A7 B1 B4 B7 B10 C1 C2 C5 F3 H4 H11 A2 A4 A9 A10 A11 A12 B3 B5 B8 B9 B12 C4 C6 C7 C8 C10 D4 D5 D6 D8 D9 D10 D12 E3 E5 E10 F4 F7 F11 F12 G3 G5 G8 G9 G10 H3 H5 H10 H12 A3 A5 A6 A8 B2 B6 B11 C3 C9 C11 C12 D1 D2 D3 D7 D11 E1 E2 E4 E6 E7 E8 E9 E11 E12 F1 F2 F5 F6 F8 F9 F10 G1 G2 G4 G6 G7 G11 G12 H1 H2 H6 H7 H8 H9
B1 D–Galactonic Acid Lactone
B2 D–Galactonic Acid Lactone
A1 a–Hydroxybutyric Acid
A2 a–Hydroxybutyric Acid
A1 a–Keto Valeric Acid
A2 a–Keto Valeric Acid
B1 L–Alanyl–glycine
B2 L–Alanyl–glycine
A1 Glycyl–L–Glutamic Acid
A2 Glycyl–L–Glutamic Acid
B1 D–Serine
B2 D–Serine
A1 L–Serine
A2 L–Serine
A1 D,L–a–Glycerol Phosphate
A2 D,L–a–Glycerol Phosphate
B1 N–Acetyl–D–Glucosamine
B2 N–Acetyl–D–Glucosamine
A1 a–D–Glucose
A2 a–D–Glucose
A1 Pyruvic Acid Methyl Ester
A2 Pyruvic Acid Methyl Ester
B1 D–Gluconic Acid
B2 D–Gluconic Acid
A1 p–Hydroxy Phenylacetic Acid
A2 p–Hydroxy Phenylacetic Acid
A1 Quinic Acid
A2 Quinic Acid
A1 Sebacic Acid
A2 Sebacic Acid
A1 L–Alanine
A2 L–Alanine
A1 L–Asparagine
A2 L–Asparagine
B1 L–Aspartic Acid
B2 L–Aspartic Acid
A1 L–Glutamic Acid
A2 L–Glutamic Acid
B1 Hydroxy–L–Proline
B2 Hydroxy–L–Proline
B1 Glycerol
B2 Glycerol
N–Acetyl–D–Galactosamine
i–Erythritol
D–Galactose
m–Inositol
Maltose
D–Melibiose
b–Methyl–D–Glucoside
L–Rhamnose
Glucuronamide
Thymidine
a–D–Glucose–1–Phosphate
a–Cyclodextrin
Glycogen
Adonitol
L–Arabinose
D–Arabitol
D–Cellobiose
L–Fucose
Gentiobiose
a–D–Lactose
Lactulose
D–Mannose
D–Raffinose
D–Sorbitol
Sucrose
D–Trehalose
Xylitol
Formic Acid
D–Galactonic Acid Lactone
D–Galacturonic Acid
D–Glucosaminic Acid
D–Glucuronic Acid
a–Hydroxybutyric Acid
g–Hydroxybutyric Acid
a–Keto Butyric Acid
a–Keto Valeric Acid
D–Saccharic Acid
L–Alaninamide
L–Alanyl–glycine
Glycyl–L–Aspartic Acid
Glycyl–L–Glutamic Acid
L–Leucine
L–Phenylalanine
D–Serine
L–Serine
L–Threonine
Uridine
Phenyethyl–amine
D,L–a–Glycerol Phosphate
D–Glucose–6–Phosphate
Dextrin
Tween 40
Tween 80
N–Acetyl–D–Glucosamine
D–Fructose
a–D–Glucose
D–Mannitol
D–Psicose
Turanose
Pyruvic Acid Methyl Ester
Succinic Acid Mono–Methyl–Ester
Acetic Acid
Cis–Aconitic Acid
Citric Acid
D–Gluconic Acid
b–Hydroxybutyric Acid
p–Hydroxy Phenylacetic Acid
Itaconic Acid
a–Keto Glutaric Acid
D,L–Lactic Acid
Malonic Acid
Propionic Acid
Quinic Acid
Sebacic Acid
Succinic Acid
Bromosuccinic Acid
Succinamic Acid
D–Alanine
L–Alanine
L–Asparagine
L–Aspartic Acid
L–Glutamic Acid
L–Histidine
Hydroxy–L–Proline
L–Ornithine
L–Proline
L–Pyroglutamic Acid
D,L–Carnitine
g–Amino Butyric Acid
Urocanic Acid
Inosine
Putrescine
2–Aminoethanol
2,3–Butanediol
Glycerol
